# Autologous, lentivirus‐modified, T‐rapa cell “micropharmacies” for lysosomal storage disorders

**DOI:** 10.15252/emmm.202114297

**Published:** 2022-03-17

**Authors:** Murtaza S Nagree, Tania C Felizardo, Mary L Faber, Jitka Rybova, C Anthony Rupar, S Ronan Foley, Maria Fuller, Daniel H Fowler, Jeffrey A Medin

**Affiliations:** ^1^ Department of Medical Biophysics University of Toronto Toronto ON Canada; ^2^ Department of Pediatrics Medical College of Wisconsin Milwaukee WI USA; ^3^ Rapa Therapeutics Rockville MD USA; ^4^ Department of Pathology and Laboratory Medicine Western University London ON Canada; ^5^ Juravinski Hospital and Cancer Centre McMaster University Hamilton ON Canada; ^6^ Genetics and Molecular Pathology SA Pathology at Women's and Children's Hospital North Adelaide SA Australia; ^7^ Department of Biochemistry Medical College of Wisconsin Milwaukee WI USA

**Keywords:** gene therapy, lentivirus, lysosomal storage disorders, T cells, Genetics, Gene Therapy & Genetic Disease, Organelles, Stem Cells & Regenerative Medicine

## Abstract

T cells are the current choice for many cell therapy applications. They are relatively easy to access, expand in culture, and genetically modify. Rapamycin‐conditioning *ex vivo* reprograms T cells, increasing their memory properties and capacity for survival, while reducing inflammatory potential and the amount of preparative conditioning required for engraftment. Rapamycin‐conditioned T cells have been tested in patients and deemed to be safe to administer in numerous settings, with reduced occurrence of infusion‐related adverse events. We demonstrate that *ex vivo* lentivirus‐modified, rapamycin‐conditioned CD4^+^ T cells can also act as next‐generation cellular delivery vehicles—that is, “micropharmacies”—to disseminate corrective enzymes for multiple lysosomal storage disorders. We evaluated the therapeutic potential of this treatment platform for Fabry, Gaucher, Farber, and Pompe diseases *in vitro* and *in vivo*. For example, such micropharmacies expressing α‐galactosidase A for treatment of Fabry disease were transplanted in mice where they provided functional enzyme in key affected tissues such as kidney and heart, facilitating clearance of pathogenic substrate after a single administration.

## Graphical abstract

The therapeutic potential of lentivirus (LV)‐modified, rapamycin‐conditioned CD4^+^ T cells (T‐Rapa) was evaluated. These T‐Rapa "micropharmacies" (TRaMs) can be used as next‐generation cellular delivery vehicles to treat Lysosomal Storage Disorders (LSDs) and other inherited disorders.

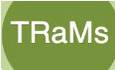

**The paper explained**

**Problem**

T cells are the state‐of‐the‐art gene carrier in many anti‐cancer gene therapy schemas. Meanwhile, hematopoietic Stem/Progenitor (HSPC)‐directed platforms show promise in clinical trials using *ex vivo* gene*‐*modified cell therapies to treat a variety of disorders such as lysosomal storage disorders (LSDs) compared to the standard‐of‐care, enzyme therapy (ET). However, some issues with HSPCs emerge that may be addressed with the use of autologous T cells, for example, the ability to isolate cells from peripheral blood without pre‐treatment, expand cells *ex vivo*, engraft with reduced immune‐ablation, and repeat administration.

**Results**

We provide proof‐of‐concept for the use of lentivirus‐modified rapamycin‐conditioned T cells—“T‐Rapa micropharmacies”—as a source of autologous enzyme production for LSDs. We modify T‐Rapa with codon‐optimized lentiviral constructs and demonstrate their ability to secrete enzymes for the correction of Fabry, Gaucher, Farber, and Pompe diseases. We show that T‐Rapa micropharmacies can be made from Fabry patient cells and that transplant of these cells into mice results in systemic reduction of pathogenic substrate.

**Impact**

T‐Rapa micropharmacies may be used to provide a continual source of enzyme compared to ET. This may alleviate some issues with conventional ET and address some difficulties of using HSPC‐directed platforms alone.

## Introduction

T cells are an established vehicle for cell and gene therapy—they can be isolated directly from a blood draw, they are easily expanded *ex vivo*, and they have the potential for extended persistence (Tebas *et al*, 2013). Various unmodified T cell products have been clinically tested, particularly in the context of anti‐cancer treatments. For example, T cells have been infused after bone marrow transplantation and patients have been treated with *ex vivo* expanded tumor infiltrating lymphocytes (Porter *et al*, 2006; Nguyen *et al*, 2019). Next‐generation immunotherapies involve genetic modification of autologous T cells to arm them with an anti‐tumor arsenal, which include engineered T cell receptors and chimeric antigen receptors (CARs) (Maude *et al*, 2014; Rapoport *et al*, 2015). The latter technology has resulted in FDA approval of T cell‐based cell and gene therapies—anti‐CD19 CAR therapies have been approved for use in adult and pediatric B cell lymphomas (Maude *et al*, 2014; Mullard, 2021). Other targets for CAR‐T cell therapies are actively being investigated, including glioblastoma, where the capacity for T cells to traffic and elicit an effect across the blood–brain barrier (BBB) has been demonstrated with venous infusion of cells (O'Rourke *et al*, 2017). The potential for *ex vivo* expansion and subsequent banking of T cells has facilitated batch‐testing and possible future re‐administration of therapeutic product (Rouce *et al*, 2015; Vairy *et al*, 2018).

Lysosomal storage disorders (LSDs) are a group of genetic deficiencies caused by absent or defective lysosomal hydrolases, leading to accumulation of substrate and lysosomal dysfunction (Fuller *et al*, 2006; Platt *et al*, 2012). LSDs often have severe pathology that can result in very early morbidity and mortality (Sun, 2018). The current standard‐of‐care for some LSDs is repeated infusion of recombinant hydrolases, termed enzyme therapy (ET; alternatively referred to as enzyme replacement therapy, or ERT) (Solomon & Muro, 2017). ET is commonly manufactured from cultures of mammalian cells engineered to overexpress the applicable enzyme (Solomon & Muro, 2017). When overexpressed, many lysosomal hydrolases are secreted from genetically modified cells and appropriately modified for potential uptake into bystander cells. ET is approved for only 10 LSDs (Garbade *et al*, 2020) but, when available, has led to increased lifespans and improved quality of life for some patients (Sun, 2018). However, this treatment modality has numerous drawbacks that can limit its use, including the following: relatively short enzyme half‐lives due to rapid clearance, only part of which is accounted for by uptake into affected cells, the need for frequent enzyme infusions, high costs of treatments, and immunogenicity against the infused product, which can further reduce efficacy (Tomatsu *et al*, 2010; Broomfield *et al*, 2016; Lenders & Brand, 2018).

Gene therapy is a promising alternative strategy for LSDs wherein autologous cells are engineered to overexpress the lysosomal enzymes (Nagree *et al*, 2019). Secreted enzyme can be taken up by non‐modified cells leading to their “cross‐correction”. Gene therapy should yield more consistent and prolonged exposure to the corrective enzyme, require less frequent treatments, and possibly induce immune tolerance (Koeberl & Kishnani, 2009; van Til *et al*, 2010; Elman *et al*, 2014). Used in over 300 clinical trials worldwide (http://www.abedia.com/wiley/vectors.php), recombinant lentiviral vectors (LVs) show favorable safety profiles and have excellent tropisms for many different cell types (Finkelshtein *et al*, 2013; Lidonnici *et al*, 2018; Marcucci *et al*, 2018). Genomic integration of proviral transgenes, introduced using LVs, ensures long‐term subsistence in all daughter cells and is associated with sustained gene expression (Kafri *et al*, 1997).

Clinical studies testing LV‐modified cell therapies show their potential to halt disease progression and treat a subset of LSD pathologies in the short term (Sessa *et al*, 2016; Khan *et al*, 2021). LV‐transduced, patient‐derived hematopoietic cells engraft following venous infusion, making them excellent vehicles to mediate systemic cross‐correction in LSDs (Sessa *et al*, 2016; Abutalib & Hari, 2017). While hematopoietic stem/progenitor cells (HSPCs) have the potential to provide life‐long correction, a bone marrow harvest or drug‐induced mobilization into the peripheral blood followed by apheresis is required to obtain sufficient cells for LV modification followed by re‐infusion (Abutalib & Hari, 2017). HSPCs are also difficult to expand *ex vivo* (Abutalib & Hari, 2017; Kumar & Geiger, 2017). Further, transduced HSPCs also require host conditioning for efficient engraftment (Abutalib & Hari, 2017; Khan *et al*, 2021); in some cases, such as with myeloablative conditioning, these protocols can be quite invasive and require extensive hospitalization (Ribeil *et al*, 2017). They may also lead to long‐term consequences. Such facets may impact patient recruitment and ultimately limit broad clinical implementation of this approach.

To avoid some of these issues, we have targeted T cells in this present study. Others have previously adapted T cell‐based platforms to produce erythropoietin, for example, for treatment of anemia (O'Neil *et al*, 2018). Specifically, we test rapamycin‐conditioned CD4^+^ T cells (T‐Rapa) here. Rapamycin conditioning reprograms the T cell metabolic state to that which is favorable for long‐term engraftment and function, including an enrichment in memory cells (He *et al*, 2011; Vodnala *et al*, 2019). Accordingly, T‐Rapa engraft better than untreated T cells in mouse transplant models (Mariotti *et al*, 2008). In patients, T_h_2‐skewed T‐Rapa successfully engraft with reduced‐intensity conditioning in allogeneic transplant settings (Fowler *et al*, 2013). Autologous T_h_1‐polarized T‐Rapa are being tested in a phase II trial (NCT04176380). Herein, we evaluate the capacity of LV‐engineered T‐Rapa to act as “micropharmacies”—cellular vehicles that can engraft and secrete enzyme into plasma for the treatment of a variety of LSDs.

## Results and Discussion

**Figure EV1:**
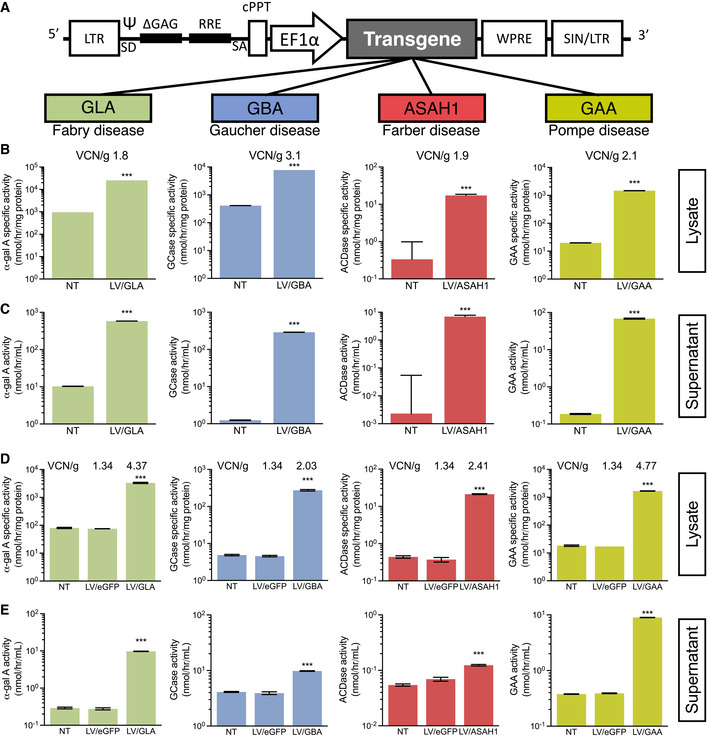
Supranormal intracellular and secreted lysosomal hydrolase enzyme activity from lentivirus‐transduced HEK293T and Jurkat cells ASchematic of the modified HIV‐1‐derived lentiviral backbone used in this study.B, CLentiviral vectors (LVs) were packaged and used to transduce HEK293T cells—vector copy number per genome (VCN/g) for each HEK293T line are indicated in (B). Enzyme activities in lysates (intracellular; B) and supernatants (secreted; C) were measured.D, ELVs were used to transduce Jurkat cells—VCN/g for each Jurkat line are indicated in (D). Enzyme activities in lysates (intracellular; D) and supernatant (secreted; E) enzyme activities were measured. Data information: Activities are reported as a mean of *n* = 3 seeded wells, error bars are standard deviation, and the *y*‐axis is logarithmic to base 10 to highlight activities in controls. Two‐tailed Student's *t*‐tests were used to compare activities in NT versus LV‐transduced cells, ***P < 0.001. Abbreviations—LTR: long‐terminal repeat; SIN/LTR: self‐inactivating LTR; SD: splice donor; SA: splice acceptor; Ψ: Retroviral Psi packaging element; ΔGAG: modified lentiviral Gag response element; RRE: Rev response element; cPPT: central polypurine tract; EF1α: elongation factor 1 α (promoter); WPRE: woodchuck hepatitis post‐translational regulatory element; GLA: α‐galactosidase A (α‐gal A); GBA: β‐glucocerebrosidase (GCase); ASAH1: acid ceramidase (ACDase); GAA: acid α‐glucosidase; NT: non‐transduced; LV: lentiviral transduced.

We previously created a LV with a codon‐optimized transgene that engineers expression of α‐galactosidase A (α‐gal A) that is currently applied in a clinical trial for the treatment of Fabry disease (NCT02800070) (Huang *et al*, 2017; Khan *et al*, 2021). For this present study, we also created analogous novel vectors with codon‐optimized transgenes that engineer expression of glucocerebrosidase (GCase), acid ceramidase (ACDase), and acid α‐glucosidase (GAA) for the potential treatment of Gaucher disease, Farber disease, and Pompe disease, respectively (Fig EV1A). All LVs packaged well under GLP conditions with titers ranging from 6–20 × 10^8^ infectious particles/ml as measured by qPCR using a 1‐copy cell line standard (Huang *et al*, 2017). We first tested expression from our vectors in HEK293T cells (Fig EV1B and C). We then evaluated our vectors in Jurkat cells, a cell line with CD4^+^ T cell‐like characteristics, to ensure such cells are inherently able to overexpress and secrete lysosomal enzymes (Fig EV1D and E). Transductions of both cell types led to supranormal levels of intracellular enzyme‐specific activity for each respective transgene product (Fig EV1B and D). Concomitantly, a substantial elevation of secreted enzyme activity was also observed from both cell types (Fig EV1C and E). Both intracellular and secreted levels of our transgene products were much higher in HEK293T cells, likely owing to their characteristically higher protein expression capability.

**Figure 1:**
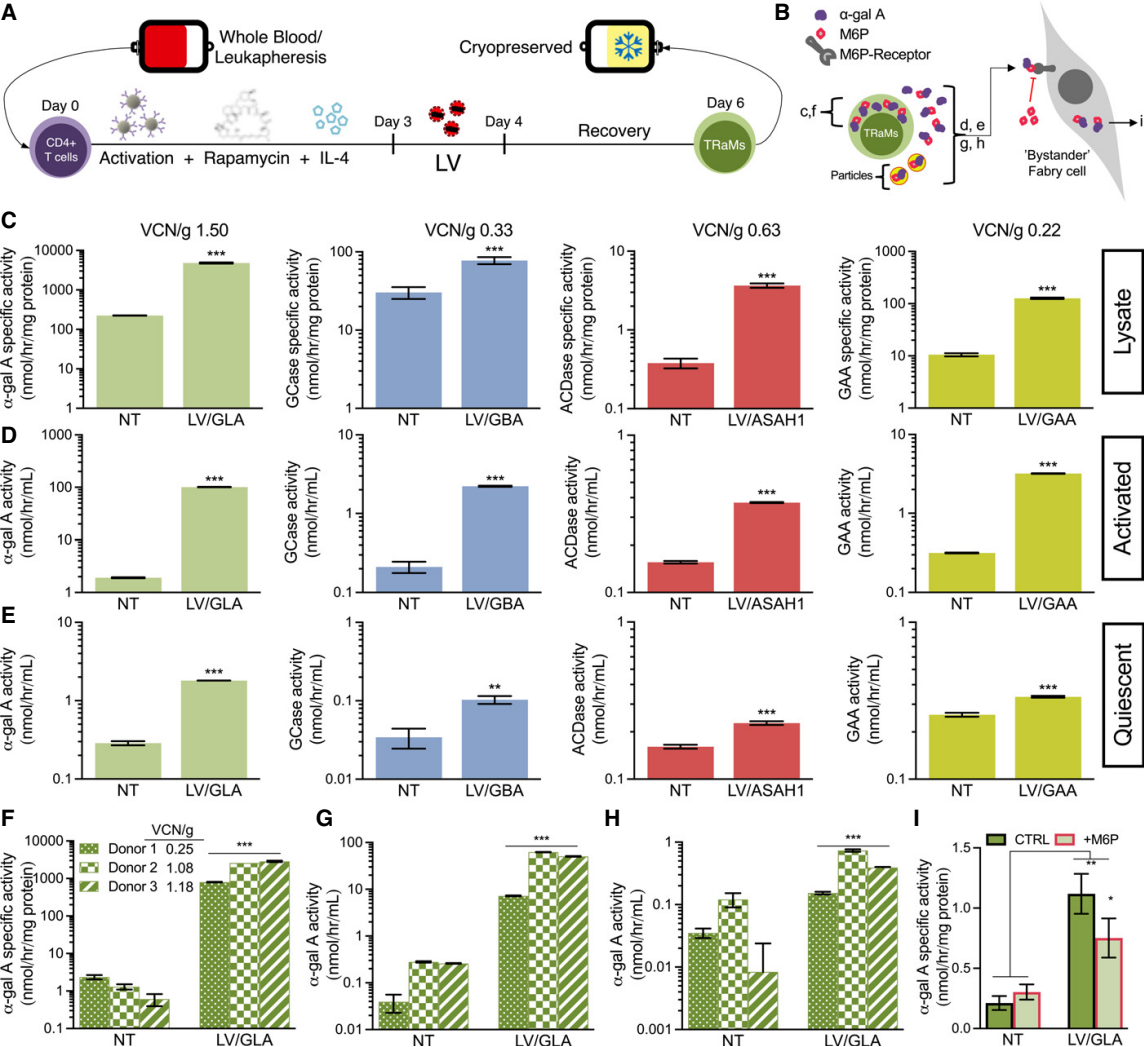
Transduced healthy (HDo) and Fabry donor (FDo)‐derived T‐Rapa “micropharmacies” (TRaMs) secrete functional lysosomal enzymes ASchematic of TRaM production. CD4^+^ T cells were enriched by positive selection from leukaphereses of multiple HDos and 3 FDos—details are provided in Materials and Methods.BEnzyme compartments that are measured in C‐I and Fig EV4G; intracellular, total secreted and colloidal particles (enclosed in red and yellow circles).CHDo‐TRaMs were engineered with vectors relevant to treatment of Fabry, Gaucher, Farber, and Pompe diseases. Intracellular lysosomal enzyme specific activities were measured in transduced T‐Rapa and controls. Transductions varied across vectors/donors, as indicated.D, EEnzyme activity was assessed in transduced T‐Rapa supernatants compared to controls in activated/dividing (D) and quiescent/resting (E) states.FCells from all FDo were stably transduced, as indicated. Intracellular α‐galactosidase A (α‐gal A)‐specific activities were determined.G, HSecreted α‐gal A activity was measured from FDo‐derived TRaMs in activated (G), and quiescent/resting (H) states.Iα‐Gal A‐specific activity in Fabry patient‐derived skin fibroblasts after 6 h of exposure to conditioned media from TRaMs in the presence or absence of 1 mM M6P. Data information: Activities in (C–H) are reported as a mean of *n* = 3 seeded wells per donor, error bars are standard deviation. Data for (C–E) are representative of at least two donors. Activities were compared between matched transduced and control cells using pairwise two‐tailed Student's *t*‐tests, ***P < 0.001 and **P < 0.01. Activities in (I) are reported as a mean of *n* = 3 independent treatments per condition, error bars are standard deviation. Two‐way analysis of variance with Tukey's multiple comparison test was used to compare activities, **P < 0.01 and *P < 0.05. Abbreviations—VCN/g: vector copy number per genome; GLA: α‐gal A; GBA: β‐glucocerebrosidase (GCase); ASAH1: acid ceramidase (ACDase); GAA: acid α‐glucosidase; NT: non‐transduced; LV: lentiviral transduced; CTRL: vehicle control.

**Figure EV2:**
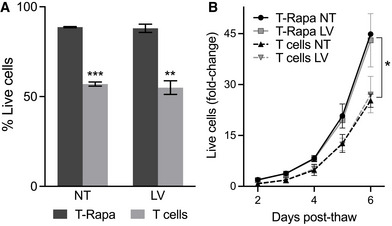
Rapamycin‐conditioned T cells are resistant to cryogenic stress, regardless of transduction Viability of rapamycin‐conditioned T cells was compared to control T cells after a freeze–thaw cycle by trypan blue exclusion.Rapamycin‐conditioned or control cells were thawed and seeded for culture with CD3/CD28 T‐Activator beads. Live cells, as determined by trypan blue exclusion, were counted every day. Data information: Viability in (A) is shown as a mean of *n* = 3 donor cells, error bars are standard error of the mean. Two‐tailed Student's *t*‐test was used to compare viability between rapamycin‐conditioned (T‐Rapa) and control cells, ***P < 0.001 and **P < 0.01. For comparison, data in (B) is presented as mean of fold‐change from original seeding number of *n* = 2 donors each with *n* = 2 replicates, error bars are the range obtained from samples from two donors. Two‐way analysis of variance with Bonferroni's multiple comparison test showed significantly higher fold‐change of live cells at days 5 and 6, *P < 0.05. Abbreviations—NT: non‐transduced; LV: lentivirus‐modified.

We have previously described our rapamycin conditioning protocol to generate a T_h_2‐polarized phenotype (Fowler *et al*, 2013). We then integrated a single transduction step to this protocol, previously optimized using a lentivirus that engineers expression of eGFP to consistently obtain at least 40% eGFP‐expressing cells (Felizardo *et al*, 2013), to generate T‐Rapa micropharmacies (TRaMs; Fig 1A). As part of our internal quality control, and, in addition to the previously studied benefits of rapamycin conditioning (Mariotti *et al*, 2008; He *et al*, 2011; Fowler *et al*, 2013), TRaMs appeared to be more resilient to cryogenic stress (Fig EV2A) and may have better potential to expand than control T cells produced without rapamycin conditioning (Fig EV2B).

We first manufactured LSD‐directed TRaMs from healthy donors (HDo). We achieved different rates of transduction for each of our four vectors despite using congruent transduction protocols (Fig 1C). However, transduction rates did not vary dramatically when T‐Rapa from different HDo (*n* = 3) were transduced with the same vector at MOIs of 30–60 (LV/GLA: 1.40–1.78 VCN/g; LV/GBA: 0.21–0.33 VCN/g; LV/GAA: 0.11–0.22 VCN/g). This suggests each vector may have its own limitations or need further optimization. However, even a lower VCN/g still led to significant intracellular enzyme expression (Fig 1C). TRaMs were able to generate considerable levels of functional secreted enzyme (Fig 1D) and maintained secretion, even when quiescent, though at a much lower level (Fig 1E). To evaluate whether primary patient‐derived T‐Rapa performed similarly, we produced and tested LV/GLA‐modified TRaMs from Fabry patient donor (FDo) cells. We observed productive transductions with high intracellular α‐gal A‐specific activities in FDo‐derived TRaMs (Fig 1F) along with α‐gal A secretion (Fig 1G) that was maintained at lower levels even when cells were quiescent (Fig 1H). α‐Gal A activity produced was lower but comparable to that found in LV/GLA‐modified HDo TRaMs.

**Figure EV3:**
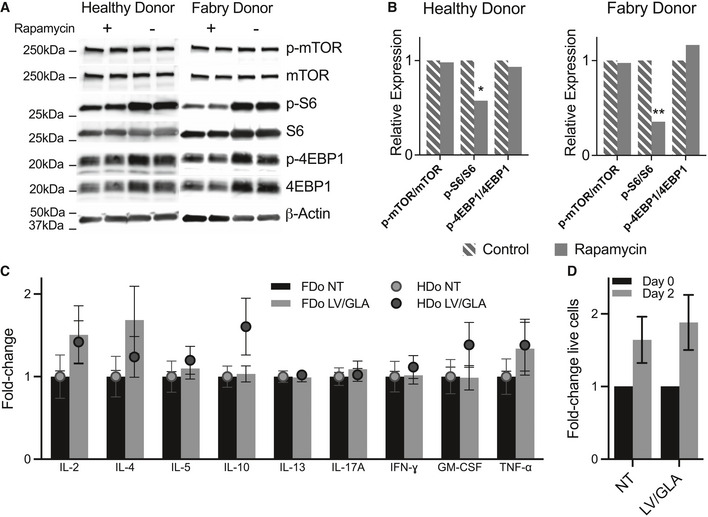
Rapamycin treatment has comparable low‐depth inhibition of the mTOR pathway in Healthy (HDo) and Fabry donor (FDo) T cells, and overexpression of α‐gal A does not suppress T cell function CD4^+^ T cells from HDo or FDo were cultured with CD3/CD28 T‐Activator beads and 1 µM rapamycin for 3 days and harvested to perform Western blots for markers of mTOR inhibition.Relative expression of phosphorylated mTOR targets were determined from densitometry of Western blots; intensities were normalized to β‐Actin, then to total protein, then to results from control cells.FDo‐ and HDo‐derived TRaMs were seeded with CD3/CD28 T‐Activator beads for 24 h and secreted cytokines were measured by a Luminex assay using a custom multiplex panel.Transduced FDo TRaMs and controls (from three independent donors) were seeded with CD3/CD28 T‐Activator beads for 2 days. Live cells were counted by trypan blue exclusion. Data information: Bars in (B) represent means of relative expression from *n* = 2 individual replicates of cells from single donors. Error bars have been omitted; values vary by 0.4–25% for the control‐treated groups, and 1–10% for the rapamycin‐treated groups. Two‐way analysis of variance with Fisher's LSD test was used to compare expression, **P < 0.01 and *P < 0.05. Fold‐changes in (C) were calculated by normalizing cytokine concentrations to the average of matched non‐transduced (NT) controls. Mean fold‐change is plotted for *n* = 3 technical replicates of 3 sets of FDo and 1 set of HDo cells, error bars are standard error of the mean. A two‐way analysis of variance with Tukey's multiple comparison test was used to compare cytokine levels between groups; no significant changes were found. Data in (D) is shown as mean fold‐change from day 0, error bars are standard error of the mean. A two‐tailed Student's *t*‐test was used to compare growth of transduced and control cells in response to short‐term stimulation. Abbreviations—TRaMs: T‐Rapa micropharmacies; LV/GLA: lentivirus transduced to express α‐galactosidase A; IL: interleukin; IFN: interferon; GM‐CSF: granulocyte‐monocyte colony‐stimulating factor; TNF: tumor necrosis factor.

We used cytokine production following CD3/CD28 bead‐based activation as an indicator of T cell function. First, our rapamycin treatment led to similar, partial inhibition of the signaling pathway downstream of mTORC1 when comparing FDo‐ and HDo‐derived T cells, as indicated by partial reduction of S6 phosphorylation relative to total levels, but unchanged phospho‐4EBP1 (Fig EV3A and B). Cytokine production was not suppressed due to transgene overexpression in HDo or FDo TRaMs (Fig EV3C). Similarly, the immediate growth response following exposure to activating beads was not suppressed in the transduced population (Fig EV3D). Taken together, these data suggest TRaMs can express and secrete lysosomal enzymes while maintaining these facets of normal T cell function.

**Figure EV4:**
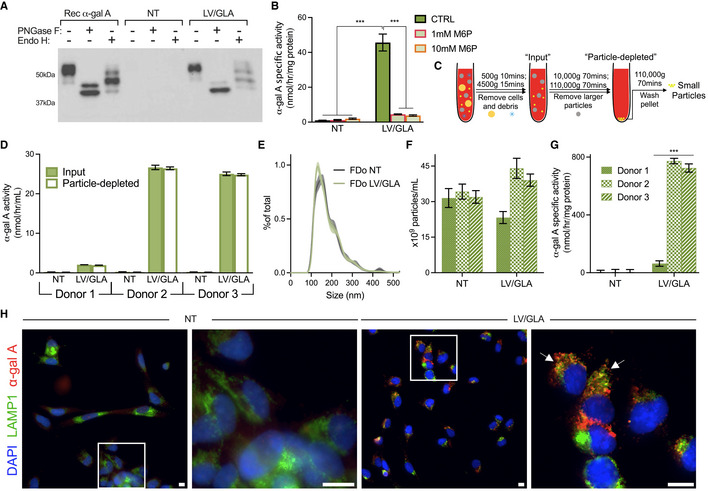
LV/GLA secreted enzyme is mature, predominantly soluble, and can be taken up by Fabry fibroblasts ASupernatants from culture of NT and LV/GLA HDo‐TRaMs, and recombinant α‐gal A were digested with the indicated glycosidases and Western blot with anti‐α‐gal A antibody was performed.Bα‐Gal A specific activity in Fabry patient‐derived skin fibroblasts after 6 h of exposure to conditioned media from HDo‐TRaMs in the presence or absence of 1 and 10 mM M6P.CSchematic of the series of centrifugation steps used to clear FDo‐TRaM conditioned media of particulates.DEnzyme activity was measured in conditioned media before (input) and after (particle‐depleted) ultracentrifugation.E, FNanoparticle tracking analysis was used to measure size distribution (E) and concentration (F) of colloidal particles collected after the final ultracentrifugation.Gα‐Gal A specific activities in colloidal particles isolated from conditioned media by ultracentrifugation were determined.HImmunofluorescent microscopy of Fabry patient‐derived fibroblasts after culture with NT or LV/GLA 293T conditioned supernatant. For each condition, a representative image is shown with enlarged section from white box to the right. White arrows indicate cells in which enzyme has been taken up and is co‐localized with lysosomal staining. Data information: Activities in (B) are reported as a mean of *n* = 3 independent treatments per condition, error bars are standard deviation. Activities in (B) were compared using a two‐way analysis of variance with Tukey's multiple comparison test. Activities in (D) are reported as a mean of *n* = 3 depletions per donor, error bars are standard deviation. Particle‐depleted activities in (D) were normalized to input activities and compared using a one‐way analysis of variance with Tukey's multiple comparison test. Concentrations of particles of each size were converted to a percentage of total particles for comparison between donors in (E); data are plotted as a distribution of means of *n* = 3 replicates of 3 donors for each of NT and LV/GLA, shown with a dark gray or green line. Standard error of the mean is shown as shading extending around these lines. Distributions were compared using a two‐way analysis of variance. Data in (F) is plotted as means of *n* = 3 counts for each donor, with standard error of the mean. NT and LV/GLA concentrations were compared using a one‐way analysis of variance with Tukey's multiple comparison test. Activities in (G) are reported as a mean of *n* = 3 isolations for each donor, error bars are standard deviation. Activities were compared between particles isolated from matched transduced and control cells using pairwise two‐tailed Student's *t*‐tests, ***P < 0.001. Scale bars in (H) represent 10 µm. Abbreviations—TRaMs: T‐Rapa micropharmacies; HDo and FDo: Healthy and Fabry patient‐derived cells, respectively; GLA: α‐galactosidase A (α‐gal A); NT: non‐transduced; LV/GLA: lentivirus transduced to express α‐galactosidase A; CTRL: vehicle control; PNGase F: Peptide‐*N*‐Glycosidase F; Endo H: Endoglycosidase H; DAPI: 4',6‐diamidino‐2‐phenylindole; LAMP1: Lysosomal‐associated membrane protein 1.

As the N‐linked oligosaccharide status of human α‐gal A can affect proper protein folding, secretion, and enzymatic activity (Matsuura *et al*, 1998), we assessed the maturity of LV/GLA TRaM secreted α‐gal A. Western blot analyses were performed on media from control NT and LV/GLA‐modified HDo‐TRaMs digested with PNGase F and Endo H. The deglycosylation patterns were similar to that of CHO‐cell‐produced recombinant α‐gal A (Fig EV4A) and consistent with literature reports (Yasuda *et al*, 2020; Pagant *et al*, 2021) indicating that TRaM‐produced α‐gal A from LV/GLA is appropriately glycosylated.

Soluble lysosomal enzymes can be taken up by cells via receptor‐mediated endocytosis mechanisms including the mannose‐6‐phosphate (M6P) receptor (Solomon & Muro, 2017). To assess whether α‐gal A secreted from TRaMs could be taken up *in vitro,* we treated FDo‐derived skin fibroblasts with supernatant from FDo‐derived TRaMs for a short period of time (Fig 1B). Baseline α‐gal A activity of these FDo fibroblasts was about 1% of that found in wild‐type fibroblasts. An increase in intracellular activity was found after treatment with TRaM supernatant, and 33% less α‐gal A activity was observed in the presence of soluble M6P (Fig 1I). This suggests that some level of cross‐correction can be achieved using FDo‐TRaMs and that the correction is partially achieved by uptake via the M6P receptor but may also be achieved by other pathways. FDo‐derived skin fibroblast intracellular α‐gal A activity was also increased when treated with supernatant from HDo‐TRaMs (Fig EV4B). This activity was reduced by greater than 90% with the addition of 1 and 10 mM M6P. To determine if FDo‐TRaM secreted enzyme activity was from *bona fide* soluble enzyme, and not solely from microvesicles, for example, we depleted TRaM culture supernatants of colloidal particles by ultracentrifugation (Fig EV4C). Almost all (95–99%) of the α‐gal A activity was retained in TRaM culture supernatants after particle depletion (Fig EV4D). Interestingly, colloidal particles isolated from TRaM culture supernatant, ranging in size from 90–350 nm (Fig EV4E and F), still possessed a significant amount of α‐gal A‐specific activity (Fig EV4G), though this activity in particles comprised a very low level (2–4%) of total supernatant activity when normalized to volume. Activity in particles may be expected as some extracellular vesicles such as exosomes are formed via the endolysosomal pathway and may thus carry lysosomal enzymes (van Niel *et al*, 2018).

Cross‐correction of cells requires that TRaM‐produced α‐gal A be taken up and trafficked to the lysosome. Only background fluorescence for α‐gal A is exhibited in immortalized Fabry patient fibroblasts. After culture with supernatant from NT‐HEK293T cells, no increase in α‐gal A fluorescence is detected, but is detected with supernatant from LV/GLA‐modified HEK293T cells where it is co‐localized with the lysosomes (Fig EV4H). These data suggest therapeutic enzyme is secreted from TRaMs in various forms and capable of facilitating cross‐correction at low levels after a short exposure *in vitro*.

**Figure 2:**
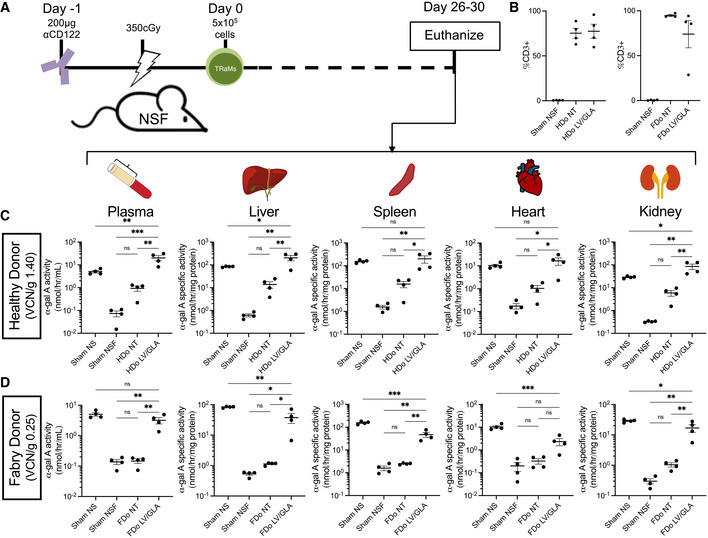
TRaMs result in elevated enzyme expression following xenotransplantation ASchematic of the transplant protocol optimized for T‐Rapa cells. NOD/SCID/Fabry (NSF) mice were conditioned and TRaMs were administered intravenously—details are indicated in Materials and Methods. Mice were euthanized 4 weeks post‐infusion.BEngraftment was evaluated by flow cytometry for human CD3 expression.C, Dα‐Gal A activity in plasma, and specific activities in liver, spleen, heart, and kidneys were measured after transplant of transduced healthy donor (HDo)‐derived (C) or Fabry donor (FDo)‐derived (D) TRaMs, non‐transduced (NT) cells, or in sham‐treated NOD/SCID (NS) and NSF mice. Data information: %CD3 of lymphocyte‐gated parent population in (B) and enzyme activities in (C, D) are reported as values for individual mice (*n* = 4) with a line indicating the mean, and error bars showing standard error of the mean. The y‐axis is logarithmic to base 10 in (C, D) to highlight activities in controls. One‐way analyses of variance with Tukey's multiple comparison tests were used to compare activities between groups, ***P < 0.001, **P < 0.01, *P < 0.05, and ns *P* > 0.05. Abbreviations—TRaMs: T‐Rapa micropharmacies; LV/GLA: lentivirus transduced to express α‐gal A; VCN/g: vector copy number per genome.

Next, we postulated that consistent exposure to α‐gal A secreted from transduced TRaMs over a longer period of time may result in a functional benefit *in vivo*. We thus evaluated the ability of TRaMs to cross‐correct using a xenograft model (Fig 2A). This model utilizes immunocompromised mice lacking α‐gal A activity (NOD/SCID/Fabry; NSF) (Pacienza *et al*, 2012). Various doses (0.1–2.5 million cells) of unmodified HDo‐derived T‐Rapa were xenografted in a pilot study. We determined that the minimal dose of 0.5 million cells provided successful engraftment. In addition, we found that mice were moribund between 4–6 weeks post‐engraftment—as such we ended our experiment after 4 weeks. Sham‐treated mice were also moribund after 4–6 weeks—we thus attributed this observation to the conditioning protocol we used. Successful engraftment was determined by end‐point flow cytometry for T cell markers in peripheral blood cells (Fig 2B). Elevated levels of α‐gal A activity were found in plasma with engraftment of either HDo‐derived (Fig 2C) or FDo‐derived (Fig 2D) TRaMs, demonstrating successful *in vivo* “dispensing” of the therapeutic factor. VCN/g of the cell products that were used are indicated. A significant increase in α‐gal A activity was observed in most tissues tested in mice given LV/GLA‐modified TRaMs, indicative of locally present enzyme (Fig 2C and D). The only exception was in hearts of mice engrafted with FDo‐derived TRaMs (Fig 2D panel 4)—though a small amount of α‐gal A activity was still present—the result was significant when only the NT and LV/GLA‐treated groups were compared (Student's *t*‐test, *P* = 0.0469). The α‐gal A activities in various tissues of mice engrafted with HDo‐derived cells was higher than or normalized to that found in NOD/SCID mice, that is, wild‐type levels (Fig 2C). Mouse models overexpressing α‐gal A show no pathology (Kase *et al*, 1998; Ashley *et al*, 2002), and pre‐clinical and clinical studies in which α‐gal A is overexpressed in hematopoietic cells report no adverse effects (Huang *et al*, 2017; Khan *et al*, 2021) suggesting increased α‐gal A is not deleterious to health. Conversely, normalization to wild‐type activity levels was not achieved in any tissues tested in mice engrafted with FDo‐derived cells (Fig 2D). This difference is likely due to the higher transduction efficiency of the HDo‐derived TRaMs and not due to the use of FDo‐derived TRaMS. In fact, a relationship between VCN/g and activity in FDo‐derived cells is evident from *in vitro* data (Fig 1F–H). Use of FDo‐derived TRaMs with higher transduction efficiency, as with Donors 2 and 3 (Fig 1F), would likely result in higher enzyme levels and potential for normalization to wild‐type levels. Nonetheless, the lower enzyme levels achieved in this experiment may be sufficient to reduce disease burden, as examined below.

**Figure 3:**
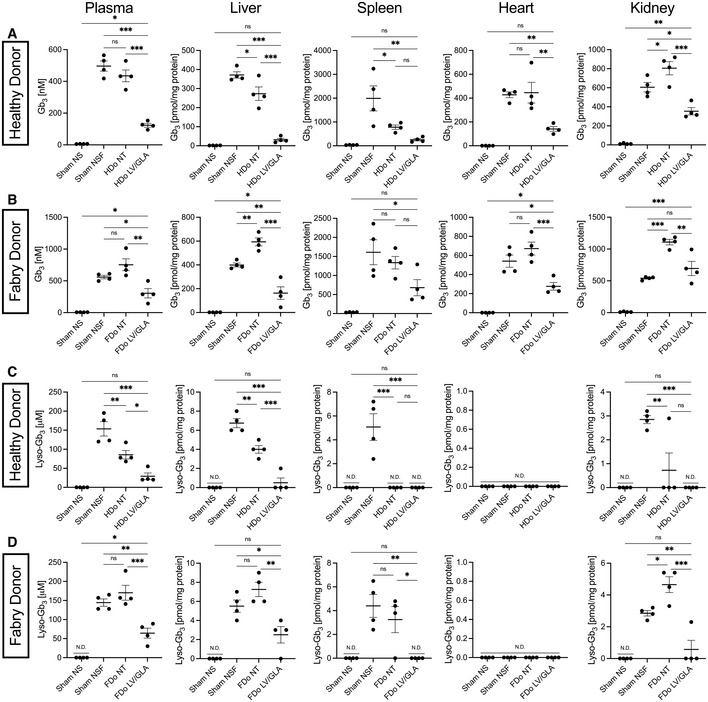
TRaMs reduce accumulated substrate following xenotransplantation A, BLevels of globotriaosylceramide (Gb_3_) were measured by LC/MS in plasma and extracts of liver, spleen, heart, and kidneys of mice engrafted with healthy donor (HDo)‐derived (A) or Fabry donor (FDo)‐derived (B) TRaMs, non‐transduced (NT) cells, or in sham‐treated NOD/SCID (NS) and NSF mice.C, DLevels of lyso‐Gb_3_ were measured by LC/MS in plasma and extracts of tissues of mice engrafted with HDo‐derived (C) or FDo‐derived (D) TRaMs. Data information: Sphingolipid concentrations are reported as values for individual mice (*n* = 4) with a line indicating the mean, and error bars showing standard error of the mean. Data from (A–D) were compared using one‐way analyses of variance with Tukey's multiple comparison tests, ***P < 0.001, **P < 0.01, *P < 0.05, and ns *P* > 0.05. Abbreviations—TRaMs: T‐Rapa micropharmacies; LV/GLA: lentivirus transduced to express α‐gal A; n.d.: not detected. Source data are available online for this figure.

Evidence of α‐gal A activity localized within tissues is not sufficient to demonstrate cross‐correction; infiltrated intraparenchymal T cells may contribute to the activity levels measured. NSF mice display highly elevated levels of globotriaosylceramides (Gb_3_), the main family of glycosphingolipids that accumulates in Fabry disease, as well as their derivative, globotriaosylsphingosine (lyso‐Gb_3_) (Pacienza *et al*, 2012). We quantified the levels of these substrates, which better demonstrates uptake and functionality of α‐gal A produced from TRaMs. We confirmed elevation of Gb_3_ (Fig 3A and B) and lyso‐Gb_3_ (Fig 3C and D) levels in NSF mice compared to NOD/SCID. Interestingly, we also observed a significant elevation in Gb_3_ levels upon engraftment of FDo‐derived unmodified T‐Rapa in liver and kidney (Fig 3B). A smaller but significant increase was also found in kidneys of mice engrafted with HDo‐derived unmodified T‐Rapa (Fig 3A). While we have not investigated the reason for this side effect, the increased Gb_3_ is not expected to be contributed by infiltrated engrafted cells, as this increase is not apparent in spleens where high levels of engrafted T cells are likely to reside (Fig 3B). Gb_3_ levels were significantly reduced from levels found in NSF mice in all organs tested after xenograft of HDo‐derived TRaMs (Fig 3A). Additionally, Gb_3_ levels in liver, spleen, and heart were not significantly different from those found in wild‐type mice (Fig 3A), suggesting a trend toward metabolic normalization.

**Figure EV5:**
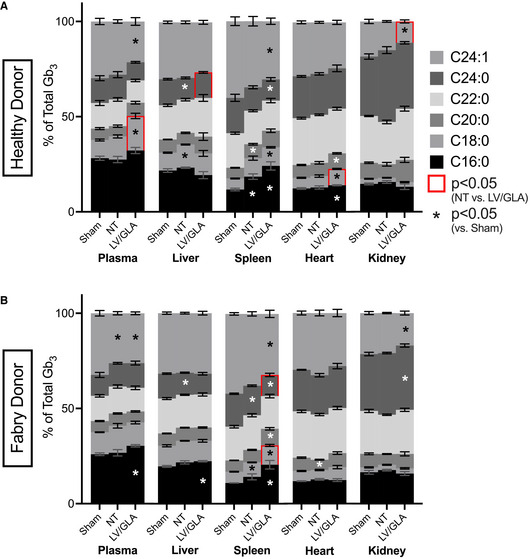
Shifts in proportions of acyl‐chain variants (ACVs) of Gb_3_ after xenotransplantation of TRaMs are small and inconsistent and suggest no species bias of transgenic α‐gal A A, BProportions of Gb_3_ ACVs as determined by LC/MS in the indicated tissues in mice transplanted with healthy donor‐derived (A) or Fabry donor‐derived (B) TRaMs compared to controls. Data information: Percentages reported are means of *n* = 4 mice, error bars are standard error of the mean. Data were compared using a two‐way analysis of variance with Tukey's multiple comparison test. Significant differences (*P* < 0.05) between ACV proportions of xenografted compared to sham‐treated mice are indicated with * in the respective bar, and those between mice xenografted with NT or LV/GLA modified T‐Rapa as a red outline. Abbreviations—TRaMs: T‐Rapa micropharmacies; LV/GLA: lentivirus transduced to express α‐galactosidase A.

FDo‐derived TRaMs were also able to significantly reduce Gb_3_ levels in plasma and in most tissues tested, when compared to sham‐treated NSF mice (Fig 3B). The reduction occurred despite a lower VCN/g, and with only a small increase in activity in the case of the heart (Fig 2D). The exception was in kidney, though Gb_3_ levels were significantly reduced in that organ back to NSF levels when compared to mice engrafted with unmodified FDo‐derived T‐Rapa (Fig 3B). Unsurprisingly, the lower VCN/g did not facilitate normalization to levels seen in NOD/SCID mice in any tissue (Fig 3B). Lyso‐Gb_3_ levels were also reduced with engraftment of either HDo or FDo‐derived TRaMs, most notably in plasma where more of this lipid appeared to be present (Fig 3C and D). Lyso‐Gb_3_ levels were normalized in most mice engrafted with TRaMs except in plasma of mice engrafted with FDo‐derived TRaMs (Fig 3C and D). These data suggest the α‐gal A produced by HDo‐derived and FDo‐derived TRaMs is taken up *in vivo* and capable of reducing accumulated substrate, despite the modest uptake levels seen *in vitro* (Fig 1I). Interestingly, engraftment of unmodified HDo‐derived T‐Rapa also significantly reduced lyso‐Gb_3_ (Fig 3C). This was also seen for Gb_3_ levels in liver (Fig 3A) suggesting the small amount of α‐gal A produced by HDo‐derived T‐Rapa (Fig 2C) is also capable of cross‐correction. We also considered the proportions of individual acyl‐chain variants (ACVs) of Gb_3_ measured in this study (Fig EV5A and B) to determine if α‐gal A produced by TRaMs had any substrate biases. In general, no dramatic or consistent shifts were seen, except in the spleen (Fig EV5A and B). As the cell milieu in the spleen is expected to be comprised largely of T cells after xenograft, the shift in ACV content is not surprising there.

Recent studies have indicated that gene therapies directing expression in a cell type‐specific manner may provide greater cumulative exposure to therapeutic enzyme and appear to ameliorate anti‐ET immune responses (Koeberl & Kishnani, 2009; van Til *et al*, 2010; Elman *et al*, 2014). *Ex vivo* engineering facilitates cell‐specific expression while also preventing direct patient exposure to vector. Herein, we have used a sequential testing pipeline to determine TRaM utility for the treatment of numerous LSDs. Despite lower transduction efficiencies with some of our vectors, we were able to demonstrate supranormal enzyme activities *in vitro*. These levels may be sufficient to correct many LSDs. Further optimization, or inclusion of elements such as cell surface markers (Wang *et al*, 2011; Scaife *et al*, 2013) or those that confer drug resistance (Jonnalagadda *et al*, 2013) may improve efficacy of TRaMs. Even without these measures, cell dosages may be adjusted to administer a fixed VCN, or cell products re‐administered, such that a desired functional outcome can be achieved, as done with other FDA‐approved T cell therapies (Rouce *et al*, 2015; Vairy *et al*, 2018).

We have demonstrated that α‐gal A secreted from TRaMs facilitates cross‐correction *in vitro* and that HDo and FDo‐derived TRaMs have potential for short‐term *in vivo* efficacy for Fabry disease. Unfortunately, our xenograft model does not permit extensive observation of engrafted mice to determine TRaM persistence, nor provide a comprehensive picture of the safety of TRaMs. Long‐term efficacy and toxicology may be determined using a different model in which multiple doses can be provided, and wherein a competitive immune environment is present, for example using syngeneic mouse‐to‐mouse engraftments using transgenic mouse lines, as mouse T cells are not readily transduced with LVs. Similarly, accurate comparison of TRaMs to other treatment types for LSDs, such as ET or HSPC‐directed gene therapies, need to be carried out in syngeneic transplant models. However, comparing our data to previous studies in which FDo‐derived CD34^+^ cells were xenografted into NSF mice (Huang *et al*, 2017), for example, suggests TRaMs are capable of eliciting comparable if not superior outcomes. Finally, Fabry mice display relatively mild phenotypes only in old animals (Shen *et al*, 2015), and our NSF model, like the parent NOD/SCID strain, it is derived from, rarely survives past 14 months. As such, the ability of TRaMs to resolve clinically relevant pathology in LSDs remains to be studied.

The exact forms in which lysosomal enzymes are secreted from TRaMs—for example, post‐translational modifications, or the nature of the particles in which they are packaged—remains to be studied. Correction in the central nervous system also remains to be examined, though here we have limited our studies to treatment of disorders with substantial visceral pathology. We have also only examined expression of unmodified lysosomal enzymes—TRaMs may also be used to deliver proteins engineered for enhanced uptake in specific tissues or to cross the blood–brain barrier, especially for treatment of neuronopathic LSDs (Okuyama *et al*, 2019). TRaMs may also serve as “recycling centers” for circulating substrates that can be taken up by engrafted cells. When TRaM production is scaled up, batch quality metrics can be established using *in vitro* data from a pipeline such as ours to predict corrective potential for individual lots of cells for each disease, as has been done in other contexts (Fraietta *et al*, 2018). TRaMs provide a source of cells that can be readily collected, manufactured, and banked. They may provide for a stand‐alone cell‐based treatment administered with no or minimal conditioning. TRaMs have the potential to persist longer than conventional protein‐based therapies but may be re‐administered as necessary for optimal treatment. TRaMs may therefore fulfill multiple roles in treatments for LSDs (and other indications).

## Materials and Methods

### Cell lines and primary cells

HEK293T/17 and Jurkat cells (clone E6‐1) were purchased from ATCC (CRL‐11268 and TIB‐152). Primary skin fibroblasts from an affected Fabry patient and related healthy control were obtained from the Coriell Institute (GM02775 and GM02270) and immortalized by introduction of SV40 large T antigen and TERT (Viral Vector Core, Blood Research Institute, Versiti, WI USA). HDo CD4^+^ T cells were isolated by positive selection using magnetic‐activated cell sorting (MACS; Miltenyi Biotec 130‐045‐101) per the manufacturer's instructions from leukapheresis packs (PPA Research 10‐0001 or StemCell Technologies 70500) after centrifugation over a Ficoll‐Paque PLUS (GE Healthcare 17144002) density barrier. FDo peripheral blood mononuclear cells (PBMCs) were enriched from discarded (i.e., the CD34^+^‐depleted) fraction of de‐identified clinical cell product (Khan *et al*, 2021) by density centrifugation using NycoPrep 1.077 solution (Axis‐Shield 1114550). CD4^+^ T cells were obtained from PBMCs by positive selection for CD4 using magnetic‐activated cell sorting (MACS) as with HDo cells.

### Cell culture

HEK293T cells were maintained in Dulbecco's modified Eagle's medium (DMEM; Gibco 11995‐065) supplemented with 10% v/v heat‐inactivated fetal bovine serum (FBS; 10437‐028 or 26140‐079, lot‐matched), 100 U/ml penicillin, 100 µg/ml streptomycin, and 2 mM l‐glutamine (1xPSQ; Gibco 10378‐016). Cells were passaged every 2 or 3 days, or at 90–95% confluency. Jurkat cells were maintained in RPMI‐1640 media (Sigma‐Aldrich R8758) supplemented with 10% v/v FBS and 1xPSQ. Jurkat cells were subcultured by dilution every 2–3 days. Immortalized skin fibroblasts were maintained in DMEM supplemented with 10% v/v FBS and 1xPSQ and were passaged every 3–5 days or when 85–90% confluency was achieved. Cells for immunofluorescence were cultured in chamber slides (Thermo Scientific 154526). All cells were maintained at 37°C with relative humidity of ~ 95%, atmospheric O_2_, and 5% CO_2_. Cell counts were determined using an automated cell counter (Countess II FL, Invitrogen) according to manufacturer's instructions; dead cells were excluded by trypan blue staining (Gibco 15250‐061).

### Plasmid construction

Transgene expression cassettes were designed based on the annotated coding sequences (CDS) from mRNA sequences in the NCBI Reference Sequence database (accession numbers: NM_000169 for GLA; NM_000157 for GBA; NM_177924 for ASAH1; NM_000152 for GAA). The CDS were codon‐optimized for improved expression in human cells and to eliminate internal restriction enzyme recognition sites. The mammalian Kozak consensus sequence (GCCACC) was included immediately upstream of the start codon, and DNA was synthesized and inserted into the pUC57 cloning vector (Genscript). Expression cassettes were subcloned into a self‐inactivating, HIV‐1‐based, lentiviral packaging plasmid backbone (pDY; Fig EV1A) produced in our laboratory, described previously (Huang *et al*, 2017). All plasmids for virus preparation were propagated in XL10‐gold ultracompetent cells (Agilent 200314) and purified using a gigaprep kit (Invitrogen K210009). Plasmid sequences were verified by DNA sequencing.

### Lentivirus packaging

Lentivirus was packaged in HEK293T cells and concentrated by ultracentrifugation essentially as described previously (Huang *et al*, 2017). Pellets were resuspended in plain X‐VIVO 20 media (Lonza 04‐448Q) in a final volume corresponding to a 2,000‐fold concentration of the viral supernatant harvested. Concentrated lentivirus was aliquoted and cryopreserved at −80°C.

### Vector copy number assay

Genomic DNA (gDNA) was harvested from 1–2 × 10^6^ cultured cells, or 200–300 µl of RBC‐lysed (Qiagen 158902) whole blood using a Puregene kit (Qiagen 158388) per manufacturer's instructions. gDNA concentration was measured using a NanoDrop One spectrophotometer. Vector copy number (VCN) was determined essentially as described previously (Huang *et al*, 2017). Briefly, TaqMan reagents were used to carry out a duplex real‐time PCR to detect WPRE, a virus‐specific element, and ACTB as a loading control. Real‐time PCR was carried out on a ViiA 7 instrument (Applied Biosystems). Normalized Ct values for transduced samples were used to infer VCN from a standard curve of known VCN/g.

### Viral titer and transduction

Productive viral titers were determined by transducing HEK293T cells with serial dilutions of virus stocks in the presence of 8 µg/ml protamine sulfate (Sigma‐Aldrich P4020). After at least three passages, gDNA was extracted and VCN/g determined as described above. The titer was calculated by inferring original vector copies per volume before dilution for each transduction.

HEK293T cells were transduced at a confluency of ~ 60–80% at an MOI of ~ 2–3. Jurkat cells were transduced by at a density of 1 × 10^6^ cells/ml at an MOI of ~5. Transductions were carried out in 1 ml/10 cm^2^ complete media with 8 µg/ml protamine sulfate. Media was replaced 16–24 h later, and cells were cultured for at least 8 days before analyses.

### T‐Rapa micropharmacy generation and culture

T‐Rapa were produced, cultured, and transduced essentially as described previously (Felizardo *et al*, 2013). Briefly, T cells were conditioned with 1 µM rapamycin (sirolimus oral solution, Pfizer) for 3 days with the following culture conditions: X‐VIVO 20 media supplemented with 5% v/v human AB serum (Gemini bio‐products 100‐512, various lots), 20 U/ml of interleukin (IL)‐2 (Roche 11147528001), 1,000 U/ml of IL‐4 (R&D Systems 204‐IL), and CD3/CD28 T‐Activator beads (Gibco 111.32D) at a 3:1 bead‐to‐cell ratio. T‐Rapa were transduced for 16–24 h after washing off rapamycin at an MOI of 30–60 in the presence of 8 µg/ml protamine sulfate. Cells were cryopreserved after 2 days of recovery (Fig 1A). Cryopreservation medium was made by making a 1:1 mixture of complete T cell culture medium and pentastarch freezing mix, comprised of 120 mg/ml pentastarch solution (Preservation Solutions Inc. PST002), 75mg/ml human serum albumin (Flexbumin 25%, Shire 2G0212), and 10% v/v dimethylsulfoxide (DMSO; Sigma‐Aldrich D1435).

Cryopreserved cell products generated above were thawed for *in vitro* studies and seeded in X‐VIVO 20 media with 5% v/v human AB serum and 4–20 U/ml of IL‐2 at 1–1.5 million cells/ml with CD3/CD28 T‐Activator beads at a bead:cell ratio of 2.5–3:1. Cells were monitored and counted daily for viability and density, and fresh media added as necessary to maintain the cultures at ~ 1 million cells/ml. After the cultures stopped dividing, seen consistently at 8 days after thaw, cells were depleted of remaining beads and used to determine VCN, as well as seeded for other studies.

### Western blotting

Components of mTORC1 signaling were analyzed in CD4^+^ T cells isolated from HDo or FDo were conditioned with 1 µM rapamycin or vehicle for 3 days as above. Cells were harvested and lysed in RIPA buffer (2017) with 1mM ethylenediamine tetraacetate (EDTA; Fisher BP120), 2 mM sodium fluoride (Sigma‐Aldrich S6776), protease (Thermo Scientific 78429), and phosphatase (Thermo Scientific 78420) inhibitors. Protein concentration of clarified lysates was determined using a biscincinoic acid (BCA) assay kit (Pierce 23225) as recommended by the manufacturer. Equal amounts of protein were separated by electrophoresis on a 4–15% gradient SDS–polyacrylamide gel (Bio‐Rad 4568083). The glycosylation status of T‐Rapa produced α‐gal A was determined in media samples from NT and LV/GLA‐modified HDo TRaMs. Equal volumes of media (and Chinese hamster ovary cell (CHO)‐produced‐recombinant α‐gal A as control) were digested with Endo H (New England Biolabs P0702) or PNGase F (New England Biolabs P0709) according to the manufacturer's instructions and separated by electrophoresis on an 8% SDS–polyacrylamide gel. Proteins were wet transferred 1.5 h or overnight to polyvinylidene fluoride membranes with a pore size of 0.2 µm (EMD Millipore ISEQ09120). Membranes were rinsed in water and stained using 0.5% w/v Ponceau S (ACROS Organics 161470100), then blocked for 1 h in 5% w/v non‐fat milk (Nestle Carnation). The following primary antibodies (Cell Signaling Technology) were used: anti‐phospho‐Ser2448‐mTOR (clone D9C2, 5536T, 1:1,000); anti‐mTOR (clone 7C10, 2983T, 1:1,000); anti‐phospho‐Thr37/46‐4EBP1 (clone 236B4, 2855T, 1:1,000); anti‐4EBP1 (clone 53H11, 9644T, 1:1,000); anti‐phospho‐Ser235/236‐S6 (clone D57.2.2E, 4858T, 1:2,000); anti‐S6 (clone 5G10, 2217S, 1:1,000); anti‐α‐gal A (clone EP5828(2), abcam 168341, 1:1,000). All antibodies were used at dilutions and conditions recommended by the manufacturer. An anti‐rabbit IgG‐HRP secondary antibody (Sigma‐Aldrich A6154) was used in 5% milk at a dilution of 1:5,000. Protein bands were detected using HRP‐reactive chemiluminescent reagent (Bio‐Rad 1705060) and imaged with a ChemiDoc MP (Bio‐Rad). For mTORC1 signaling analysis, β‐Actin was used as a loading control (clone AC‐15, Sigma‐Aldrich A3854, 1:10,000). ImageJ (v1.51) was used for densitometry, and band intensities obtained were normalized to intensities of β‐Actin, then to intensities of respective total protein.

### Media conditioning and cell lysis for enzyme studies

Transduced HEK293T cells were seeded for 2 days in appropriate complete media for a final confluence of ~ 95%. Transduced Jurkat cells were seeded at a density of 4 × 10^5^ cells/ml in appropriate complete media for 3 days. T‐Rapa were seeded at 1 × 10^6^ cells/ml after the 8‐day culture described above in appropriate complete media with CD3/CD28 T‐Activator beads at a bead:cell ratio of 1:1 and 20 U/ml IL‐2 for 24 h (activated culture), or at 1.5 × 10^6^ cells/ml with only 4 U/ml IL‐2 for 2 days (quiescent culture). HEK293T were harvested by scraping, and Jurkat and T‐Rapa by resuspension. Conditioned media was separated and clarified by centrifugation. Cell pellets were resuspended in: citrate‐phosphate buffer (CPB; Sigma‐Aldrich P4809) with 5mg/ml sodium taurocholate (CHB; Sigma‐Aldrich T4009) at pH 4.5 for α‐galactosidase A (α‐gal A) or glucocerebrosidase (GCase) activity; CHB at pH 4.0 for acid α‐glucosidase (GAA) activity; or 0.2 M sucrose with protease inhibitors for acid ceramidase (ACDase) activity. Cell lysis was carried out by 5 cycles of freeze–thaw, and lysates were clarified by centrifugation. Supernatants and lysates were both stored at −80°C.

### Enzyme assays

Fluorometric enzyme assays were modified from previously described methods (Medin *et al*, 1996a, 1996b; Okumiya *et al*, 2006; Bedia *et al*, 2010). Substrates used for α‐gal A, GCase, and GAA activities were 5 mM 4‐methylumbelliferyl‐α‐d‐galactopyranoside (Sigma‐Aldrich M7633), 15 mM 4‐methylumbelliferyl‐β‐d‐glucopyranoside (Sigma‐Aldrich M3633), and 1.5 mM 4‐methylumbelliferyl‐α‐d‐glucopyranoside (Sigma‐Aldrich M9766), respectively. Reactions were carried out in 0.1 M CPB at pH 4.0–4.5. Non‐specific activities were excluded for α‐galactosidase B and non‐lysosomal α‐amylase using 100 mM *N*‐acetyl‐d‐galactosamine (Sigma‐Aldrich A2795) and 10 µM acarbose (Sigma‐Aldrich A8980), respectively. ACDase activity was determined using 30 µM RBM14‐12 substrate (RUBAM, IQAC‐CSIC) in 20 mM sodium acetate (Fisher Scientific, BP334) buffer at a pH of 4.5. Enzyme reactions were carried out in opaque microtiter plates for 1–3 h at 37°C. α‐Gal A, GCase, and GAA reactions were stopped using 0.1 M glycine solution at pH 10; fluorescence was measured with excitation at 360nm and emission at 450 nm (Varioskan LUX, Thermo Scientific). ACDase reactions were stopped by sequential addition of 50 µl methanol and 100 µl 0.1 M glycine with 2.5 mg/ml sodium periodate (Sigma‐Aldrich 311448) at a pH of 10.6, followed by a 2‐h incubation at 37°C. Fluorescence there was measured with excitation at 355 nm and emission at 460 nm. The concentrations of reaction products were quantified by interpolation from a calibration curve. Specific activity is reported as nmol of product per hour per total protein, as for cell or tissue lysates. Protein concentration was quantified using a BCA assay kit. Otherwise, activity is reported per volume, as for supernatants and plasma.

### Cytokine profiling

T‐Rapa cells were seeded at 1 × 10^6^ cells/ml in appropriate complete media with CD3/CD28 T‐Activator beads at a bead:cell ratio of 1:1 and 20 U/ml IL‐2 for 24 h. Culture supernatants were clarified and a custom ProcartaPlex 9‐plex (Invitrogen) assay was used, as recommended by the manufacturer, to measure concentrations of the reported cytokines. The sample plates were read using a Luminex 200 instrument per manufacturer's directions with a lower bound of 100 beads per sample per cytokine. Cytokine levels of transduced T‐Rapa were normalized to matching non‐transduced control for comparison between various donors.

### Colloidal particle depletion

Transduced T‐Rapa or control cells were seeded with CD3/CD28 T‐Activator beads and maintained in culture for 6–8 days. Culture supernatants were subjected to a series of centrifugations at 4°C to deplete various particulates (Fig EV4A) essentially as described previously (Thery *et al*, 2006). Briefly, cells and large debris were removed by centrifugation, and supernatant was subjected to sequential ultracentrifugation in a swinging bucket rotor (SW 32 Ti) rotor in an Optima L‐100 XP centrifuge (Beckman Coulter) for 70 min at: 10,000 *g* to remove apoptotic bodies and dense particulates; then 110,000 *g* to deplete smaller colloidal particles. Culture supernatants before and after ultracentrifugation were collected to determine soluble α‐gal A activity. Small particulates obtained after ultracentrifugation were washed with PBS, resuspended in CHB, and lysed for α‐gal A activity analysis, as described above. Nanoparticle tracking analysis was used to confirm presence of colloidal particles, as well as their concentration and size distribution. Diluted particles were infused using a syringe pump (Harvard Apparatus 98‐4730) into a NanoSight NS300 (Malvern Panalytical). Instrument parameters were adjusted using a pooled sample, and all data acquired with identical settings. Frequencies of each particle size were averaged from technical replicates and converted to percentage of total particles to normalize data for plotting. Supernatants and particles were stored at −80°C.

### α‐Galactosidase A uptake assay

Immortalized Fabry patient‐derived skin fibroblasts were used to measure α‐gal A uptake—the assay used was adapted from previously described methods (Medin *et al*, 1996b). Culture media was conditioned by culturing transduced T‐Rapa for 2 days and added to fibroblasts buffered with Hepes with or without 1 and 10 mM mannose‐6‐phosphate (M6P; Sigma‐Aldrich M3655). Following a 6‐h incubation in regular culture conditions, media was aspirated, and cells washed and harvested by scraping in cold PBS. Cell pellets were lysed in CHB as described above. Clarified lysates were used to determine specific α‐gal A activity, as described.

### Immunofluorescence

Media on immortalized Fabry patient fibroblasts in chamber slides was changed to 80% conditioned media from control or LV/GLA‐modified HEK293T cells supplemented with 20% fresh complete media. After 6 h, the cells were fixed in ice‐cold methanol for 15 min at 4°C, then permeabilized with 0.2% Triton X‐100 in PBS for 5 min and blocked in 10% FBS in PBS for 1 h at room temperature. The cells were incubated overnight at 4°C with primary antibodies anti‐LAMP‐1 (Santa Cruz Biotechnology, clone H4A3, sc‐20011, 1:100) and anti‐α‐gal A (abcam, clone EP5828(2), 168341, 1:50) diluted in blocking buffer at dilutions recommended by the manufacturer. After PBS washes (5 × 5 min), cells were labeled for 1 h at 37°C with Alexa Fluor 488 goat anti‐mouse and Alexa Fluor 594 goat anti‐rabbit secondary antibodies (Thermo Scientific A11001, 1:750; R37117, 2 drops/ml, respectively). After PBS washes cells were mounted with Vectashield Antifade Mounting Medium (Vector Laboratories, H1000) which contains 4′,6‐diamidino‐2‐phenylindole (DAPI) to counterstain cell nuclei.

The fluorescent stained images, red and green, were captured using an AxioImager (Carl Zeiss, Germany) upright microscope using 20× or 40× Plan‐apo‐lenses. GFP/Alexa 488 was imaged with Filter set 37 (EX‐BP 450/50, BS‐FT480, Em‐BP 510/50), RFP/Alexa 594 was imaged using Filter set 20 (EX‐BP 546/12, BS‐FT560, Em‐BP 575–640) and DAPI was captured with Filter set 34 (EX‐BP 390/22, BS‐FT420, Em‐BP 460/50). Axiovision software and an Axiocam HRm 1.4MP color camera (Carl Zeiss, Germany) were used to record images with fixed exposure time for each fluorophore based on positive controls.

### T‐Rapa xenografts

NSF mice were used, along with appropriate WT controls, when 7–9 weeks old in this study (Pacienza *et al*, 2012). Mice were bred and housed at MCW's Biomedical Resource Center. Mice were provided chlorinated water and a standard autoclaved diet *ad libitum* and were switched to water containing 0.1 mg/ml enrofloxacin (Enroflox 100, Norbrook Laboratories, LTD) at least 5 days prior to starting experiments. All animal work was conducted per protocols approved by MCW's IACUC. Natural killer (NK) cells were depleted in these animals by administering 200 µg of anti‐CD122 antibody (clone TM‐β1, BioLegend 93448) by intraperitoneal injection 1 day before engraftment. Mice were further conditioned by administering 350cGy of gamma radiation (Gammacell 40 Exactor, Cesium‐137 source, Best Theratronics). Freshly thawed T‐Rapa were washed in PBS and were administered intravenously via the tail vein following a 3–5‐h recovery period. Mice were euthanized by CO_2_ asphyxiation. Blood was collected via cardiac puncture and transferred to K_2_EDTA tubes (BD 365974). A short cardiac perfusion was performed. Tissue was harvested, rinsed in PBS and immediately frozen on dry ice. Blood was processed by centrifugation at 1,000 *g* for 10 min at room temperature for plasma. Plasma and tissues were stored at −80°C long term. Blood was also processed for gDNA extraction (as above) and flow cytometry analyses (see following).

### Flow cytometry

Antibody concentrations and staining conditions were optimized for 100–150 μL of whole blood or 1–1.5 million cells. Following RBC lysis (BioLegend 420301), cells were washed once in staining buffer (1% FBS, 1 mM EDTA in PBS) and stained with FITC‐conjugated anti‐human CD3 (clone SK7, BioLegend 344804 at 1:100) and APC‐conjugated anti‐human CD4 (clone SK3, BioLegend 344614 at 1:100). Flow cytometry was performed on a FACS Calibur instrument (BD biosciences). Data were analyzed using FlowJo (v9.2 Tree Star Inc.). Leukocytes were gated for FITC and APC double‐positive populations, which reflect xenografted human CD4^+^ T cells.

### Tissue homogenization

Frozen tissue was thawed on ice in homogenization buffer: 20 mM Tris–HCl at a pH of 7.0, 0.5 M sodium chloride, and 0.1% IGEPAL with protease inhibitors. Either 0.5 mm zirconium oxide beads (Next Advance ZROB05) for liver, spleen, and kidney, or a mix of 0.5 mm and 0.9–2.0 mm steel beads (Next Advance SSB05 and SSB14B) for heart were added. Tissue was homogenized with a Next Advance Bullet Blender Storm 24 (BBY24M). One half of the homogenate was removed and diluted 5–20‐fold with CPB, freeze–thawed three times, and clarified to make lysates, as described above, for α‐gal A activity assay. The remaining homogenate was diluted five‐fold in homogenization buffer and further processed for total lipid extraction. Clarified lysates and homogenates were stored at −80°C.

### Globotriaosylceramide (Gb_3_) and lyso‐Gb_3_ quantification

The concentrations of six individual acyl‐chain variants of Gb_3_, namely C16:0, C18:0, C20:0, C22:0, C24:0, and C24:1, and lyso‐Gb_3_ were quantified by liquid chromatography electrospray ionization tandem mass spectrometry (LC‐ESI‐MS/MS) from crude tissue homogenates (0.1 mg total protein) or plasma as previously described (Saville *et al*, 2017; Talbot *et al*, 2017). Total Gb_3_ was estimated by summation of the concentrations of each individual variant measured.

### Statistical analyses

GraphPad Prism (v8.3.1 for MacOS, GraphPad Software, LLC) was used to perform statistical tests and to plot data. Most *in vitro* experiments were conducted with a minimum number of replicates (*n* = 3). *In vivo* studies were initially conducted with *n* = 4 successfully engrafted mice. Exclusion of non‐grafted mice was a pre‐established criterion. Mice of same sex were pooled in cages randomly after conditioning. Mice were randomly selected to receive a particular treatment which was administered by a blinded individual. Mice were provided IDs unrelated to their treatment, and all processing and analyses of tissue activity and Gb_3_ were conducted blindly. Our sample size (*n* = 4) gave us adequate power assuming that the difference in plasma α‐gal A activity and Gb_3_ levels between sham‐treated NS and NSF mice reflect maximum effect size. Two‐tailed, Student's *t*‐tests were used for comparing two parameters. One‐way analysis of variance (ANOVA) with Tukey's multiple comparisons test was used to compare two conditions out of multiple groups. Two‐way ANOVA with Dunnett's multiple comparisons test was used for comparing one group to another over a time course. Two‐way ANOVA with Tukey's multiple comparisons test was used when comparing multiple groups each with multiple matched variables. Statistical tests used for each experiment to report significance are indicated in figure legends, and *P* values are reported using asterisks in the style of the American Psychological Association (ns *P* > 0.05, *P < 0.05, **P < 0.01, and ***P < 0.001).

## Author contributions

**Murtaza S Nagree:** Conceptualization; Data curation; Formal analysis; Investigation; Visualization; Methodology; Writing—original draft; Writing—review and editing. **Tania C Felizardo:** Investigation; Methodology. **Mary L Faber:** Investigation; Methodology; Writing—review and editing. **Jitka Rybova:** Investigation. **C Anthony Rupar:** Investigation. **S Ronan Foley:** Resources. **Maria Fuller:** Investigation; Methodology. **Daniel H Fowler:** Conceptualization; Resources; Investigation; Project administration; Writing—review and editing. **Jeffrey A Medin:** Conceptualization; Resources; Supervision; Funding acquisition; Writing—original draft; Writing—review and editing.

**For more information**

- NORD: https://rarediseases.org
- LDN: https://lysosomaldiseasenetwork.org
- Fabry disease: https://www.omim.org/entry/301500
- Gaucher disease: https://www.omim.org/entry/230800
- Pompe disease: https://www.omim.org/entry/232300
- Farber disease: https://www.omim.org/entry/228000

## Supporting information



Source Data for Figure 3

## Data Availability

This study includes no data deposited in external repositories.
