# Supplementary material for: Autologous, lentivirus‐modified, T‐rapa cell “micropharmacies” for lysosomal storage disorders
Source: EMBO Mol Med. 2022 Mar 17;14(4):e14297. doi: 10.15252/emmm.202114297 (PMC8988206; doi:10.15252/emmm.202114297)
Supplement: Supplementary file 2 [file EMMM-14-e14297-s001.pdf]

EMBO Molecular Medicine

Autologous, Lentivirus-Modified, T-Rapa Cell "Micropharmacies" for Lysosomal Storage Disorders

Murtaza Nagree, Tania Felizardo, Mary Faber, Jitka Rybova, Tony Rupar, S Foley, Maria Fuller, Daniel Fowler, and Jeffrey Medin

DOI: 10.15252/emmm.202114297

Corresponding author: Jeffrey Medin (jmedin@mcw.edu)

Review Timeline:

Submission Date:	18th Mar 21
Editorial Decision:	14th Apr 21
Revision Received:	26th Jan 22
Editorial Decision:	4th Feb 22
Revision Received:	7th Feb 22
Accepted:	9th Feb 22

Editor: Zeljko Durdevic

Transaction Report:

(Note: With the exception of the correction of typographical or spelling errors that could be a source of ambiguity, letters and reports are not edited. Depending on transfer agreements, referee reports obtained elsewhere may or may not be included in this compilation. Referee reports are anonymous unless the Referee chooses to sign their reports.)

14th Apr 2021

Dear Dr. Medin,

Thank you for the submission of your manuscript to EMBO Molecular Medicine. We have now received feedback from the three reviewers who agreed to evaluate your manuscript. As you will see from the reports below, the referees acknowledge the interest of the study but also raise important criticism that should be addressed in a major revision.

Further consideration of a revision that addresses reviewers' concerns in full will entail a second round of review. EMBO Molecular Medicine encourages a single round of revision only and therefore, acceptance or rejection of the manuscript will depend on the completeness of your responses included in the next, final version of the manuscript. For this reason, and to save you from any frustrations in the end, I would strongly advise against returning an incomplete revision.

We would welcome the submission of a revised version within three months for further consideration. However, we realize that the current situation is exceptional on the account of the COVID-19/SARS-CoV-2 pandemic. Please let us know if you require longer to complete the revision.

I look forward to receiving your revised manuscript.

Yours sincerely,

Zeljko Durdevic

Zeljko Durdevic
Editor
EMBO Molecular Medicine

***** Reviewer's comments *****

Referee #1 (Comments on Novelty/Model System for Author):

Proof of concept paper provides abundance of data that TRAMS can serve as micropharmacoies to deliver ERT for Fabry Disease model of NOD SCID mouse Short life span of the NOD SCID model precludes long term comparison to ERT in the Fabry mouse. Difficulty of producing TRAMs in murine T-cells also limits long term comparison. Nonetheless, it's an important paper.

Referee #1 (Remarks for Author):

Novel, important clinically, well designed expts, methods well described, data nicely presented, conclusions justified.
I like the model and congratulate the authors. However, I wonder whether the Fabry mouse could be tolerized to the NT FB DO T cells by in utero infections , in which case the tolerized mouse could be treated with LV/GLA T-Cells and might allow long term response and direct comparison to ERT. That could be be a home run as a preclinical experiment.
See. for example
In utero hematopoietic cell transplantation: induction of donor specific immune tolerance and postnatal transplants.
Peranteau WH.
Front Pharmacol. 2014 Nov 12;5:251. doi: 10.3389/fphar.2014.00251. eCollection 2014.
PMID: 25429269 Free PMC article. Review.
Also
Sci Transl Med
. 2020 Feb 26;12(532):eaay8980. doi: 10.1126/scitranslmed.aay8980.

Tolerance induction and microglial engraftment after fetal therapy without conditioning in mice with Mucopolysaccharidosis type VII

Referee #2 (Comments on Novelty/Model System for Author):

The current study by Nagree and colleagues provide proof-of concept for the use of lentivirus modified rapamycin conditioned T-cells "TRaMs" as a source of autologous enzyme production for lysosomal storage disease (LSM). The study elegantly demonstrates patient derived TRaMs can be modulated with the codon-optimized lentiviral construct to express and secrete enzymes essential for the mitigation of the LSM. The use of these micropharmacies extends beyond the LSD to other diseases. The study is well designed and controlled. However, despite the ability of these LV's to secrete the target enzyme i.e GLA and reduce accumulation of Gb3 isoforms in most tissues, it is not clear if this is sufficient to resolve key clinical parameters of Fabry diseases. Moreover, it is not clear what is the toxicological impact of the basal GLA expression.

Referee #2 (Remarks for Author):

It is best suited for reports

Referee #3 (Comments on Novelty/Model System for Author):

This is a very important study in the evolving field of genetherapy approaches to treat lysosomal storage diseases. The others performed excellent experiments and adequate models, used a novel approach (at least for Fabry disease.

Referee #3 (Remarks for Author):

T cells are the current choice for many cell therapy applications. Rapamycin-conditioning ex vivo reprograms T cells, increasing their memory properties and capacity for survival, while reducing inflammatory potential and the amount of preparative conditioning required for engraftment. Rapamycin-conditioned T cells have been tested in patients and deemed to be safe to administer in numerous settings, with reduced occurrence of infusion-related adverse events. In the current study, the authors demonstrate that ex vivo lentivirus-modified, rapamycin-conditioned CD4+ T cells may also be used as next-generation cellular delivery vehicles , which they called "micropharmacies" - to disseminate corrective enzymes for lysosomal storage disorders. They evaluated the therapeutic potential of this treatment platform for Fabry, Gaucher, Farber, and Pompe diseases in vitro and in vivo. As an example, such micropharmacies expressing α-galactosidase A for treatment of Fabry disease were transplanted in mice where they provided functional enzyme in key affected tissues such as kidney and heart, facilitating clearance of pathogenic substrate after a single administration after 4 weeks.
I complement the authors for this very well conducted study. The study is well designed, performed and written. However, I have some major concerns, which I urgently invite the authors to address to improve the current data and to emphasize their study from others.

Cross correction of AGAL-deficient cells:
In Fabry disease cross correction is hard to achieve. Otherwise, affected females would not suffer from AGAL deficiency, too. In addition to lionization, the maturation of the secreted AGAL molecule is important. If the immature form (without M6P residue) is secreted, no or a decreased uptake occurs leading. Although the authors addressed this shortly within the discussion, I recommend performing some experiments analyzing the glycosylation pattern of secreted AGAL from transduced T cells. Appropriate western blots with preceding digestions with EndoH and PNGaseF should be sufficient to give us more information on the glycosylation pattern of secret AGAL. Furthermore, these experiments should easily demonstrate the proportion of immature and mature protein within the supernatant (if present).
The authors blocked AGAL uptake using 1mM M6P, demonstrating only a reduction by ~30%. Although I agree that there are possible further mechanisms involved in AGAL uptake, the chosen concentration of M6P seems to be a little low. 10 mM M6P might be more appropriate for these kinds of experiments.
AGAL uptake and localization:
Although the authors demonstrate increased intracellular AGAL activities after cellular uptake and also decreasing Gb3 concentrations in vivo, the most important question was not addressed, which is the lysosomal localization of AGAL after uptake. Thus, the authors should perform some additional experiments using immunofluorescence imaging to demonstrate the presence of AGAL in lysosomes of cross-corrected cells. These experiments do not have to be performed in mice for sure, but rather than in AGAL-deficient cell lines such as HEK, etc.

Minor:

Since this study focus on LSD, I would recomment to use only the term and abbreviation for enzyme replacement therapy (ERT) istead of ET, which is more exactly in this very field.

Department of Pediatrics
Hematology/Oncology/BMT

Date: January 7, 2022

To: Dr. Zeljko Durdevic
Scientific Editor
EMBO Molecular Medicine

Re: EMM-2021-14297
Letter Submission: Autologous, Lentivirus-Modified, T-Rapa Cell "Micropharmacies" for Lysosomal Storage Disorders

Dear Dr. Durdevic-

We have carefully examined the Reviewers' comments provided to us for our Letter submission to EMBO Molecular Medicine (EMM-2021-14297). We have also taken some extra time to address important questions raised by the Reviewers (thank you for allowing us this concession). We are returning the manuscript after making a number of revisions. Comments by Reviewers are in black type and point-by-point responses are highlighted in red below:

Referee #1 (Comments on Novelty/Model System for Author): Proof of concept paper provides abundance of data that TRAMS can serve as micropharmacoies to deliver ERT for Fabry Disease model of NOD SCID mouse Short life span of the NOD SCID model precludes long term comparison to ERT in the Fabry mouse. Difficulty of producing TRAMs in murine T-cells also limits long term comparison. Nonetheless, it's an important paper.

(Remarks for Author): Novel, important clinically, well designed expts, methods well described, data nicely presented, conclusions justified. I like the model and congratulate the authors. However, I wonder whether the Fabry mouse could be tolerized to the NT FB DO T cells by in utero infections , in which case the tolerized mouse could be treated with LV/GLA T-Cells and might allow long term response and direct comparison to ERT. That could be be a home run as a preclinical experiment.

See. for example In utero hematopoietic cell transplantation: induction of donor specific immune tolerance and postnatal transplants. Peranteau WH. Front Pharmacol. 2014 Nov 12;5:251. doi: 10.3389/fphar.2014.00251. eCollection 2014. PMID: 25429269 Free PMC article. Review.

Also

Jeffrey A. Medin, PhD
MACC Fund Professor, Departments of Pediatrics and Biochemistry
Vice Chair of Research Innovation, Department of Pediatrics
Research Director, Division of Pediatric Hematology/Oncology/BMT
Director GMP Vector Lab

8701 Watertown Plank Road. CRI C4540
Milwaukee, WI 53226-0509 (414) 955-4118 jmedin@mcw.edu

MEDICAL COLLEGE OF WISCONSIN

Department of Pediatrics
Hematology/Oncology/BMT

Sci Transl Med 2020 Feb 26;12(532):eaay8980. doi: 10.1126/scitranslmed.aay8980. Tolerance induction and microglial engraftment after fetal therapy without conditioning in mice with Mucopolysaccharidosis type VII

Response: We thank the Reviewer for their positive comments and for recognizing the potential of our work. We also thank the Reviewer for their interesting suggestion and accompanying references – the idea certainly warrants further investigation. As we understand, the references provided seem to address intraspecies transplantation, i.e., mouse-to-mouse transplantation, where HLA (or H2 in mice) mismatch may preclude adult transplantation, but recipients could be tolerized to such mismatch prior to development of their adaptive immune system. The ability to facilitate interspecies transplant, i.e., human-to-mouse, would indeed be a home run in the cell therapy field. We are aware of few previous attempts to transplant human immune cells into immunocompetent mice *in utero* for tolerization, for example:

> Gore, M. M., Kolhapure, R. M., Govardhan, M. K., & Banerjee, K. (1993). Xenogenic transfer of human lymphocytes in tolerized mice. *Immunology*, *80*(4), 664–665.

Note that tolerization is unlikely to provide a benefit in our immunocompromised model used in this study, however, as there is no adaptive immune system (the mice are in the NOD/SCID background) to affect. Other groups have attempted "humanizing" immunocompromised mice - i.e., transplanting them with a human hematopoietic system at a young age – to provide a more 'balanced' environment for transplanted immune cells. We are, however, not aware of any studies showing that this would promote longevity of the transplant or increase the lifespan of the transplanted mice. Finally, we feel that developing such ground-breaking techniques are outside of the scope of this Letter submission.

Referee #2 (Comments on Novelty/Model System for Author): The current study by Nagree and colleagues provide proof-of concept for the use of lentivirus modified rapamycin conditioned T-cells "TRaMs" as a source of autologous enzyme production for lysosomal storage disease (LSM). The study elegantly demonstrates patient derived TRaMs can be modulated with the codon-optimized lentiviral construct to express and secrete enzymes essential for the mitigation of the LSM. The use of these micropharmacies extends beyond the LSD to other diseases. The study is well designed and controlled. However, despite the ability of these LV's to secrete the target enzyme i.e GLA and reduce accumulation of Gb3 isoforms in most tissues, it is not clear if this is sufficient to resolve key clinical parameters of Fabry diseases. Moreover, it is not clear what is the toxicological impact of the basal GLA expression. (Remarks for Author): It is best suited for reports

We thank the Reviewer for their comments and agree that this study does not address whether the levels

Jeffrey A. Medin, PhD
MACC Fund Professor, Departments of Pediatrics and Biochemistry
Vice Chair of Research Innovation, Department of Pediatrics
Research Director, Division of Pediatric Hematology/Oncology/BMT
Director GMP Vector Lab

8701 Watertown Plank Road. CRI C4540
Milwaukee, WI 53226-0509 (414) 955-4118 jmedin@mcw.edu

of enzyme produced are sufficient to resolve clinical parameters in these disorders. Even our clinical LV-mediated trial in Fabry disease does not do that (Khan et al, Nat Comm 2021).

> Khan, A., Barber, D.L., Huang, J., Rupar, C.A., Rip, J.W., Auray-Blais, C., Boutin, M., O'Hoski, P., Gargulak, K., McKillop, W.M., Fraser, G., Wasim, S., LeMoine, K., Jelinski, S., Chaudhry, A., Prokopishyn, N.L., Morel, C.F., Couban, S., Duggan, P.R., Fowler, D.H., Keating, A., West, M.L., Foley, R., & Medin, J.A. (2021). Lentivirus-mediated gene therapy for Fabry disease. *Nat Comm.* 12:1178.

Also, as known in the field, Fabry mouse models do not present with any dramatic phenotypes related to pathology seen in patients due to their Gb_3 accumulation. Subtle phenotypes that have been quantified usually present in very old animals, as demonstrated by Shen et al. (2015). Our NSF model, like the parent NOD/SCID strain it is derived from, rarely survives longer than 14 months. <u>This is an important point to clarify – as such, we have modified our text to highlight this point in our Discussion as follows:</u>

> <u>Pg 15</u>: Finally, Fabry mice display relatively mild phenotypes only in old animals (Shen et al, 2015), and our NSF model, like the parent NOD/SCID strain it is derived from, rarely survives past 14 months. As such, the ability of TRaMs to resolve clinically relevant pathology in LSDs remains to be studied.

This reference has also been added to the bibliography.

We also acknowledge that we do not present thorough studies in this Letter format to assess any toxicity of GLA overexpression. However, we defer to previous studies by our group (many) and others regarding this. For example, two models of transgenic mice in which GLA is systemically overexpressed to very high levels have no known pathology (Ashley et al., 2002; Kase et al., 1998).

> Ashley, G. A., Desnick, R. J., Gordon, R. E., & Gordon, J. W. (2002). High overexpression of the human alpha-galactosidase A gene driven by its promoter in transgenic mice: implications for the treatment of Fabry disease. *Journal of investigative medicine : the official publication of the American Federation for Clinical Research*, *50*(3), 185–192.

> Kase, R., Shimmoto, M., Itoh, K., Utsumi, K., Kotani, M., Taya, C., Yonekawa, H., & Sakuraba, H. (1998). Immunohistochemical characterization of transgenic mice highly expressing human lysosomal alpha-galactosidase. *Biochimica et biophysica acta*, *1406*(3), 260–266. https://doi.org/10.1016/s0925-4439(98)00012-x

<u>Jeffrey A. Medin, PhD</u>
MACC Fund Professor, Departments of Pediatrics and Biochemistry
Vice Chair of Research Innovation, Department of Pediatrics
Research Director, Division of Pediatric Hematology/Oncology/BMT
Director GMP Vector Lab

Department of Pediatrics
Hematology/Oncology/BMT

Other groups have overexpressed GLA using different methods, for example AAV (Yasuda et al., 2020) without reporting any toxic effects.

> Yasuda, M., Huston, M. W., Pagant, S., Gan, L., St Martin, S., Sproul, S., Richards, D., Ballaron, S., Hettini, K., Ledeboer, A., Falese, L., Cao, L., Lu, Y., Holmes, M. C., Meyer, K., Desnick, R. J., & Wechsler, T. (2020). AAV2/6 Gene Therapy in a Murine Model of Fabry Disease Results in Supraphysiological Enzyme Activity and Effective Substrate Reduction. *Molecular therapy. Methods & clinical development*, *18*, 607–619.

Further, our group has previously completed toxicology studies in mice where continuous GLA overexpression was engineered in HSPCs to direct it to all daughter hematopoietic lineages – no toxicity related to GLA expression was found (Huang et al., 2017).

> Huang, J., Khan, A., Au, B. C., Barber, D. L., López-Vásquez, L., Prokopishyn, N. L., Boutin, M., Rothe, M., Rip, J. W., Abaoui, M., Nagree, M. S., Dworski, S., Schambach, A., Keating, A., West, M. L., Klassen, J., Turner, P. V., Sirrs, S., Rupar, C. A., Auray-Blais, C., … Medin, J. A. (2017). Lentivector Iterations and Pre-Clinical Scale-Up/Toxicity Testing: Targeting Mobilized $CD34^+$ Cells for Correction of Fabry Disease. *Molecular therapy. Methods & clinical development*, *5*, 241–258.

As mentioned above, this informed a clinical trial (NCT02800070) where no deleterious effects of GLA overexpression have been observed thus far (Khan et al., 2021). Finally, as we demonstrate in this paper (Figure EV3), we found no effect of GLA overexpression on cytokine profiles of T-Rapa cells. To communicate this point to Readers we have modified our text as follows:

> Pg 11: Mouse models overexpressing α-gal A show no pathology (Ashley et al, 2002; Kase et al., 1998), and pre-clinical and clinical studies in which α-gal A is overexpressed in hematopoietic cells report no adverse effects (Huang et al., 2017; Khan et al., 2021) suggesting increased α-gal A is not deleterious to health.

These references have also been added to the bibliography. Finally, we appreciate the Reviewers' final comment which affirms our decision to submit this succinct work in a 'Letter' format.

Referee #3 (Comments on Novelty/Model System for Author): This is a very important study in the evolving field of genetherapy approaches to treat lysosomal storage diseases. The others performed excellent experiments and adequate models, used a novel approach (at least for Fabry disease.

Jeffrey A. Medin, PhD
MACC Fund Professor, Departments of Pediatrics and Biochemistry
Vice Chair of Research Innovation, Department of Pediatrics
Research Director, Division of Pediatric Hematology/Oncology/BMT
Director GMP Vector Lab

8701 Watertown Plank Road. CRI C4540
Milwaukee, WI 53226-0509 (414) 955-4118 jmedin@mcw.edu

MEDICAL COLLEGE OF WISCONSIN

Department of Pediatrics
Hematology/Oncology/BMT

(Remarks for Author):
T cells are the current choice for many cell therapy applications. Rapamycin-conditioning ex vivo reprograms T cells, increasing their memory properties and capacity for survival, while reducing inflammatory potential and the amount of preparative conditioning required for engraftment. Rapamycin-conditioned T cells have been tested in patients and deemed to be safe to administer in numerous settings, with reduced occurrence of infusion-related adverse events.

In the current study, the authors demonstrate that ex vivo lentivirus-modified, rapamycin-conditioned CD4+ T cells may also be used as next-generation cellular delivery vehicles , which they called "micropharmacies" - to disseminate corrective enzymes for lysosomal storage disorders. They evaluated the therapeutic potential of this treatment platform for Fabry, Gaucher, Farber, and Pompe diseases in vitro and in vivo. As an example, such micropharmacies expressing α-galactosidase A for treatment of Fabry disease were transplanted in mice where they provided functional enzyme in key affected tissues such as kidney and heart, facilitating clearance of pathogenic substrate after a single administration after 4 weeks.

I complement the authors for this very well conducted study. The study is well designed, performed and written. However, I have some major concerns, which I urgently invite the authors to address to improve the current data and to emphasize their study from others.

We sincerely thank the Reviewer for their thorough reading of our manuscript, their positive comments, and their very helpful suggestions to strengthen our submission.

Cross correction of AGAL-deficient cells:
In Fabry disease cross correction is hard to achieve. Otherwise, affected females would not suffer from AGAL deficiency, too.

We agree with the Reviewer that cross-correction is difficult to achieve in Fabry disease. This point was made clearly in the fine commentary by Beck and Cox in 2019.

> Beck M and Cox TM (2019) Comment: Why are females with Fabry disease affected? *Mol Genet Metab Rep 21*: 100529.

We now believe that overexpression of GLA is necessary to get adequate secretion. And this secretion can occur using many different cell types as the source platform. Indeed, all of our previous work informed our clinical trial (Khan *et al*, 2021, see reference above). There, Fabry patients treated with our gene therapy still have detectable levels of GLA in circulation (one patient is now out 5 years) even with a fairly low vector copy number in their PBMCs.

Jeffrey A. Medin, PhD
MACC Fund Professor, Departments of Pediatrics and Biochemistry
Vice Chair of Research Innovation, Department of Pediatrics
Research Director, Division of Pediatric Hematology/Oncology/BMT
Director GMP Vector Lab

8701 Watertown Plank Road. CRI C4540
Milwaukee, WI 53226-0509 (414) 955-4118 jmedin@mcw.edu

Department of Pediatrics
Hematology/Oncology/BMT

In addition to lionization, the maturation of the secreted AGAL molecule is important. If the immature form (without M6P residue) is secreted, no or a decreased uptake occurs leading. Although the authors addressed this shortly within the discussion, I recommend performing some experiments analyzing the glycosylation pattern of secreted AGAL from transduced T cells. Appropriate western blots with preceding digestions with EndoH and PNGaseF should be sufficient to give us more information on the glycosylation pattern of secret AGAL. Furthermore, these experiments should easily demonstrate the proportion of immature and mature protein within the supernatant (if present).

We thank the Reviewer for this excellent suggestion. We performed the recommended experiments. We added these results to (new) Figure EV4A and modified the text on page 9 of our Letter submission. The following text was added:

> Pg 9: As the N-linked oligosaccharide status of human α-gal A can affect proper protein folding, secretion, and enzymatic activity (Matsuura *et al*, 1998) we assessed the maturity of LV/GLA TRaM secreted α-gal A. Western blot analyses were performed on media from control NT and LV/GLA-modified HDo-TRaMs digested with PNGase F and Endo H. The deglycosylation patterns were similar to that of CHO-cell-produced recombinant α-gal A (Fig EV4A) and consistent with literature reports (Pagant *et al*, 2021 and Yasuda *et al*, 2020) indicating that TRaM-produced α-gal A from LV/GLA is appropriately glycosylated.

We also appended the Methods section and bibliography appropriately.

The authors blocked AGAL uptake using 1mM M6P, demonstrating only a reduction by ~30%. Although I agree that there are possible further mechanisms involved in AGAL uptake, the chosen concentration of M6P seems to be a little low. 10 mM M6P might be more appropriate for these kinds of experiments.

Following the Reviewer's suggestion, we repeated this experiment with both 1 mM and 10mM M6P. We have added the new data to (new) Fig EV4B. We have also added new text to page 9 of our Letter submission.

> Pg 9: FDo-derived skin fibroblast intracellular α-gal A activity was also increased when treated with supernatant from HDo-TRaMs (Fig EV4B). This activity was reduced by greater than 90% with the addition of 1mM and 10mM M6P.

We also appended the Methods section appropriately.

Jeffrey A. Medin, PhD
MACC Fund Professor, Departments of Pediatrics and Biochemistry
Vice Chair of Research Innovation, Department of Pediatrics
Research Director, Division of Pediatric Hematology/Oncology/BMT
Director GMP Vector Lab

8701 Watertown Plank Road. CRI C4540
Milwaukee, WI 53226-0509 (414) 955-4118 jmedin@mcw.edu

Department of Pediatrics
Hematology/Oncology/BMT

AGAL uptake and localization:
Although the authors demonstrate increased intracellular AGAL activities after cellular uptake and also decreasing Gb3 concentrations in vivo, the most important question was not addressed, which is the lysosomal localization of AGAL after uptake. Thus, the authors should perform some additional experiments using immunofluorescence imaging to demonstrate the presence of AGAL in lysosomes of cross-corrected cells. These experiments do not have to be performed in mice for sure, but rather than in AGAL-deficient cell lines such as HEK, etc.

We agree with the Reviewer that this is an important issue. We thank the Reviewer for suggesting how we can strengthen this present manuscript submission. We have performed the specific experiment as directed. New IF data is added to (new) Figure EV4. We have also changed the text in the Results and Discussion section appropriately and appended the Methods. Page 10 of the Results and Discussion section now reads:

> Pg10: Cross-correction of cells requires that TRaM-produced α-gal A be taken up and trafficked to the lysosome. Only background fluorescence for α-gal A is exhibited in immortalized Fabry patient fibroblasts. After culture with supernatant from NT- HEK293T cells, no increase in α-gal A fluorescence is detected (Fig EV4H), but is detected with supernatant from LV/GLA-modified HEK293T cells (Fig EV4I) and is co-localized with the lysosomes (Fig EV4J).

The Reviewer's comment here has also prompted us to develop new reagents in the lab to look at this issue in detail in future studies (beyond the scope of this succinct 'Letter' submission). Another way to study this effect is to isolate lysosomes themselves. In the interim we have begun to adapt the method of Abu-Remaileh *et al*, 2017.

> Abu-Remaileh M, Wyant GA, Kim C, Laqtom NN, Abbasi M, Chan SH, Freinkman E, Sabatini DM (2017) Lysosomal metabolomics reveals V-ATPase- and mTOR-dependent regulation of amino acid efflux from lysosomes. Science 358: 807-813.

This technique allows fairly rapid isolation of lysosomes; i.e. much more comprehensive tracking and substrate localization experiments in the future. To do this we engineered expression of the human lysosome-specific Transmembrane protein-192 (TMEM192 Gene ID: 201931) fused to 3 tandem hemagglutinin (HA)-epitopes by employing a dual promoter pCCL LV vector (pCCL.TMEM-HA3x.eGFP) that we generated. Recent experiments using immortalized primary skin fibroblasts from an affected Fabry patient (i75) and related healthy control (i70) allowed rapid isolation of TMEM192-HA3x affinity labeled lysosomes following LV transductions. This approach will allow us to rapidly isolate lysosomes in future studies and determine protein and substrate content in their proper context.

Jeffrey A. Medin, PhD
MACC Fund Professor, Departments of Pediatrics and Biochemistry
Vice Chair of Research Innovation, Department of Pediatrics
Research Director, Division of Pediatric Hematology/Oncology/BMT
Director GMP Vector Lab

Department of Pediatrics
Hematology/Oncology/BMT

Minor:
Since this study focus on LSD, I would recomment to use only the term and abbreviation for enzyme replacement therapy (ERT) istead of ET, which is more exactly in this very field.

We thank the Reviewer for this comment. We are seeing the term ET much more often in the literature now and have chosen to go with that. It is also more accurate from a biochemical point of view as most patients are not null for α-gal A protein. They have protein but low activity. The enzyme is thus not being 'replaced' as much as it is being 'augmented'.

Dr. Durdevic: we thank you again for allowing us to submit this revised manuscript to EMBO Mol Med and for the additional time extensions to complete the prescribed work. We look forward to successful adjudication of our submission.

For the authors,

Jeffrey A. Medin, PhD
MACC Fund Professor, Departments of Pediatrics and Biochemistry
Vice Chair of Research Innovation, Department of Pediatrics
Research Director, Division of Pediatric Hematology/Oncology/BMT
Director GMP Vector Lab

8701 Watertown Plank Road. CRI C4540
Milwaukee, WI 53226-0509 (414) 955-4118 jmedin@mcw.edu

4th Feb 2022

Dear Dr. Medin,

Thank you for the submission of your revised manuscript to EMBO Molecular Medicine. I am pleased to inform you that we will be able to accept your manuscript pending the following final amendments:

1) In the main manuscript file, please do the following:
- Make sure that all special characters display well.
- Remove text highlight colour.
- In M&M, provide the antibody dilutions that were used for each antibody.
- In M&M, statistical paragraph should reflect all information that you have filled in the Authors Checklist, especially regarding randomization, blinding, replication etc.
2) Conflict of interest: Rename "Conflict of interest" to "Disclosure Statement & Competing Interests". We updated our journal's competing interests policy in January 2022 and request authors to consider both actual and perceived competing interests. Please review the policy https://www.embopress.org/competing-interests and update your competing interests if necessary.
3) Synopsis: Please check your synopsis text and image before submission with your revised manuscript. Please be aware that in the proof stage minor corrections only are allowed (e.g., typos).
4) As part of the EMBO Publications transparent editorial process initiative (see our Editorial at http://embomolmed.embopress.org/content/2/9/329), EMBO Molecular Medicine will publish online a Review Process File (RPF) to accompany accepted manuscripts. This file will be published in conjunction with your paper and will include the anonymous referee reports, your point-by-point response and all pertinent correspondence relating to the manuscript. Let us know whether you agree with the publication of the RPF and as here, if you want to remove or not any figures from it prior to publication. Please note that the Authors checklist will be published at the end of the RPF.
5) Please provide a point-by-point letter INCLUDING my comments as well as the reviewer's reports and your detailed responses (as Word file).

I look forward to reading a new revised version of your manuscript as soon as possible.

Yours sincerely,
Zeljko Durdevic

Zeljko Durdevic
Editor
EMBO Molecular Medicine

***** Reviewer's comments *****

Referee #2 (Comments on Novelty/Model System for Author):

The authors have addressed all concerns of this reviewer, recommend the acceptance of the manuscript and congratulate the authors for the outstanding study.

Referee #2 (Remarks for Author):

It is best suited for Reports

Referee #3 (Remarks for Author):

This is a revised version of a previously subbmitted manuscript. The authors performed a very accurately revision including additional experiments, which further substantiate their findings. All major and minor comments were satisfactorily answered and I complement the authors again for this excellent work. Furthermore, I would like to thank them for sharing their unpublished data and methods with me.

Comments by Reviewers after review of revised manuscript are in black text with our responses in red:

Referee #2 (Comments on Novelty/Model System for Author):
The authors have addressed all concerns of this reviewer, recommend the acceptance of the manuscript and congratulate the authors for the outstanding study.

Response: We thank the reviewer for taking the time to review the revised manuscript and are pleased we addressed all concerns.

Referee #2 (Remarks for Author):
It is best suited for Reports

Response: Thank you for reviewing the revised manuscript which will be published in the 'Letter' format.

Referee #3 (Remarks for Author):
This is a revised version of a previously subbmitted manuscript. The authors performed a very accurately revision including additional experiments, which further substantiate their findings. All major and minor comments were satisfactorily answered and I complement the authors again for this excellent work. Furthermore, I would like to thank them for sharing their unpublished data and methods with me.

Response: We appreciate the careful reading of our revised manuscript and our additional unpublished data and methods. We thank the Reviewer for the kind comments, and we are glad we were able to address your concerns regarding our original submission.

We are pleased to inform you that your manuscript is accepted for publication and is now being sent to our publisher to be included in the next available issue of EMBO Molecular Medicine.

EMBO PRESS

YOU MUST COMPLETE ALL CELLS WITH A PINK BACKGROUND ↓

PLEASE NOTE THAT THIS CHECKLIST WILL BE PUBLISHED ALONGSIDE YOUR PAPER

Corresponding Author Name: Jeffrey A. Medin

Journal Submitted to: EMBO Molecular Medicine
Manuscript Number: EMM-2021-14297

Reporting Checklist For Life Sciences Articles (Rev. June 2017)

This checklist is used to ensure good reporting standards and to improve the reproducibility of published results. These guidelines are consistent with the Principles and Guidelines for Reporting Preclinical Research issued by the NIH in 2014. Please follow the journal's authorship guidelines in preparing your manuscript.

A- Figures
1. Data
The data shown in figures should satisfy the following conditions:

- ➔ the data were obtained and processed according to the field's best practice and are presented to reflect the results of the experiments in an accurate and unbiased manner.
- ➔ figure panels include only data points, measurements or observations that can be compared to each other in a scientifically meaningful way.
- ➔ graphs include clearly labeled error bars for independent experiments and sample sizes. Unless justified, error bars should not be shown for technical replicates.
- ➔ if n< 5, the individual data points from each experiment should be plotted and any statistical test employed should be justified
- ➔ Source Data should be included to report the data underlying graphs. Please follow the guidelines set out in the author ship guidelines on Data Presentation.

2. Captions
Each figure caption should contain the following information, for each panel where they are relevant:

- ➔ a specification of the experimental system investigated (eg cell line, species name).
- ➔ the assay(s) and method(s) used to carry out the reported observations and measurements
- ➔ an explicit mention of the biological and chemical entity(ies) that are being measured.
- ➔ an explicit mention of the biological and chemical entity(ies) that are altered/varied/perturbed in a controlled manner.

- ➔ the exact sample size (n) for each experimental group/condition, given as a number, not a range;
- ➔ a description of the sample collection allowing the reader to understand whether the samples represent technical or biological replicates (including how many animals, litters, cultures, etc.).
- ➔ a statement of how many times the experiment shown was independently replicated in the laboratory.
- ➔ definitions of statistical methods and measures:
  - common tests, such as t-test (please specify whether paired vs. unpaired), simple χ^2 tests, Wilcoxon and Mann-Whitney tests, can be unambiguously identified by name only, but more complex techniques should be described in the methods section;
  - are tests one-sided or two-sided?
  - are there adjustments for multiple comparisons?
  - exact statistical test results, e.g., P values = x but not P values < x;
  - definition of 'center values' as median or average;
  - definition of error bars as s.d. or s.e.m.

Any descriptions too long for the figure legend should be included in the methods section and/or with the source data.

In the pink boxes below, please ensure that the answers to the following questions are reported in the manuscript itself. Every question should be answered. If the question is not relevant to your research, please write NA (non applicable). We encourage you to include a specific subsection in the methods section for statistics, reagents, animal models and human subjects.

USEFUL LINKS FOR COMPLETING THIS FORM

http://www.antibodypedia.com
http://1degreebio.org
http://www.equator-network.org/reporting-guidelines/improving-bioscience-research-repor
http://grants.nih.gov/grants/olaw/olaw.htm
http://www.mrc.ac.uk/Ourresearch/Ethicsresearchguidance/Useofanimals/index.htm
http://ClinicalTrials.gov
http://www.consort-statement.org
http://www.consort-statement.org/checklists/view/32-consort/66-title
http://www.equator-network.org/reporting-guidelines/reporting-recommendations-for-tumo
http://datadryad.org
http://figshare.com
http://www.ncbi.nlm.nih.gov/gap
http://www.ebi.ac.uk/ega
http://biomodels.net/
http://biomodels.net/miriam/
http://jjj.biochem.sun.ac.za
https://osp.od.nih.gov/biosafety-biosecurity-and-emerging-biotechnology/
http://www.selectagents.gov/

B- Statistics and general methods

Please fill out these boxes ↓ (Do not worry if you cannot see all your text once you press return)

1.a. How was the sample size chosen to ensure adequate power to detect a pre-specified effect size?	In vitro studies were conducted with a minimum number of samples (n=3). In vivo studies were conducted with an initial sample size of n=4 animals per group. Power analyses were conducted for plasma enzyme activity and Gb3 levels independently. A maximum effect size was determined by comparing levels in NS (wild-type) and NSF (Fabry) animals. Power analyses suggested a sample size of n=4 gave us maximum power.
1.b. For animal studies, include a statement about sample size estimate even if no statistical methods were used.	An initial sample size of n=4 engrafted animals was chosen. The difference in plasma levels of enzyme activity and Gb3 in NS and NSF mice were assumed to be the maximum effect size, and were used to ensure our analyses were adequately powered.
2. Describe inclusion/exclusion criteria if samples or animals were excluded from the analysis. Were the criteria pre-established?	We excluded animals in which human CD3 cells were not detected by flow cytometry in peripheral blood after euthanasia. This criteria was pre-established; animals without hCD3 were considered non-engrafted.
3. Were any steps taken to minimize the effects of subjective bias when allocating animals/samples to treatment (e.g. randomization procedure)? If yes, please describe.	Mice of same sex were pooled in cages randomly after conditioning. Mice were randomly selected to receive a treatment group. The individual injecting mice was blinded to the treatment being provided.
For animal studies, include a statement about randomization even if no randomization was used.	Mice were pooled after irradiation, and injected randomly.
4.a. Were any steps taken to minimize the effects of subjective bias during group allocation or/and when assessing results (e.g. blinding of the investigator)? If yes please describe.	Mice were provided IDs unrelated to their treatment, and all processing and analyses of tissue activity and Gb3 were conducted blindly.
4.b. For animal studies, include a statement about blinding even if no blinding was done	Investigators were blinded when determining enzyme activity and Gb3 levels.
5. For every figure, are statistical tests justified as appropriate?	Yes
Do the data meet the assumptions of the tests (e.g., normal distribution)? Describe any methods used to assess it.	We did not assess whether our data meet assumptions of various statistical tests. We use assays for which technical error is well-established and for which we have prior experience.
Is there an estimate of variation within each group of data?	Yes

Is the variance similar between the groups that are being statistically compared?	Yes

C- Reagents

6. To show that antibodies were profiled for use in the system under study (assay and species), provide a citation, catalog number and/or clone number, supplementary information or reference to an antibody validation profile. e.g., Antibodypedia (see link list at top right), 1DegreeBio (see link list at top right).	The following primary antibodies were used for western blotting. From Cell Signaling Technology: anti-phospho-Ser2448-mTOR (clone D9C2, 5536T); anti-mTOR (clone 7C10, 2983T); anti-phospho-Thr37/46-4EBP1 (clone 236B4, 2855T); anti-4EBP1 (clone 53H11, 9644T); anti-phospho-Ser235/236-S6 (clone D57.2.2E, 4858T); anti-S6 (clone 5G10, 2217S). From abcam: anti -a-gal A (clone EP5828(2), 168341). An anti-rabbit IgG-HRP secondary antibody (Sigma-Aldrich A6154) was used. An antibody against β-Actin was used as a loading control (clone AC-15, Sigma-Aldrich A3854). For flow cytometry, FITC-conjugated anti-human CD3 (BioLegend 344804, clone SK7) and APC-conjugated anti-human CD4 (BioLegend 344614, clone SK3) were used. The following antibodies were used for immunofluorescence. From abcam: anti -a-gal A (clone EP5828(2), 168341). From Santa Cruz Biotechnology: anti LAMP-1 (clone H4A3, sc-20011). Secondary antibodies were from ThermoScientific: Alexa Fluor 488 goat anti-mouse (A11011) and Alexa Fluor 594 goat anti-rabbit (R37117).
7. Identify the source of cell lines and report if they were recently authenticated (e.g., by STR profiling) and tested for mycoplasma contamination.	Cell lines were obtained from ATCC (HEK293T/17 and Jurkat Clone E6-1). Validation reports are available from the ATCC website. Cell lines were tested and found to be free of mycoplasma.

* for all hyperlinks, please see the table at the top right of the document

D- Animal Models

8. Report species, strain, gender, age of animals and genetic modification status where applicable. Please detail housing and husbandry conditions and the source of animals.	NOD/SCID mice and Fabry mice (Gla-/-) bred into the NOD/SCID background (Pacienza et al 2012) were used. Both male and female mice were used at roughly equal ratios. NOD/SCID mice were obtained from The Jackson Laboratories (Bar Harbor, Maine, USA). NOD/SCID/Fabry mice have been maintained by our laboratory using standard breeding practices. Mice in this study were maintained at the Medical College of Wisconsin's Biomedical Resource Center per their standard practices, and were provided with environmental enrichment (Enviro-Dri), and chlorinated water and standard rodent feed (5001) ad libitum. No more than 5 mice were housed per cage, and we avoided keeping single mice in cages. Mice were given water with 0.1mg/mL enrofloxacin as a broad spectrum prophylactic antibiotic prior to irradiation.
9. For experiments involving live vertebrates, include a statement of compliance with ethical regulations and identify the committee(s) approving the experiments.	All studies and procedures involving animals were approved by the Medical College of Wisconsin's Institutional Animal Care and Use Committee (IACUC). Animal numbers were minimized. We complied with all ethical regulations.
10. We recommend consulting the ARRIVE guidelines (see link list at top right) (PLoS Biol. 8(6), e1000412, 2010) to ensure that other relevant aspects of animal studies are adequately reported. See author guidelines, under 'Reporting Guidelines'. See also: NIH (see link list at top right) and MRC (see link list at top right) recommendations. Please confirm compliance.	We have reported relevant aspects of our animal studies per the ARRIVE guidelines in the manuscript text.

E- Human Subjects

11. Identify the committee(s) approving the study protocol.	NA
12. Include a statement confirming that informed consent was obtained from all subjects and that the experiments conformed to the principles set out in the WMA Declaration of Helsinki and the Department of Health and Human Services Belmont Report.	NA
13. For publication of patient photos, include a statement confirming that consent to publish was obtained.	NA
14. Report any restrictions on the availability (and/or on the use) of human data or samples.	NA
15. Report the clinical trial registration number (at ClinicalTrials.gov or equivalent), where applicable.	NA
16. For phase II and III randomized controlled trials, please refer to the CONSORT flow diagram (see link list at top right) and submit the CONSORT checklist (see link list at top right) with your submission. See author guidelines, under 'Reporting Guidelines'. Please confirm you have submitted this list.	NA
17. For tumor marker prognostic studies, we recommend that you follow the REMARK reporting guidelines (see link list at top right). See author guidelines, under 'Reporting Guidelines'. Please confirm you have followed these guidelines.	NA

F- Data Accessibility

18: Provide a "Data Availability" section at the end of the Materials & Methods, listing the accession codes for data generated in this study and deposited in a public database (e.g. RNA-Seq data: Gene Expression Omnibus GSE39462, Proteomics data: PRIDE PXD000208 etc.) Please refer to our author guidelines for 'Data Deposition'.<br><br>Data deposition in a public repository is mandatory for:<br>a. Protein, DNA and RNA sequences<br>b. Macromolecular structures<br>c. Crystallographic data for small molecules<br>d. Functional genomics data<br>e. Proteomics and molecular interactions	NA
19. Deposition is strongly recommended for any datasets that are central and integral to the study; please consider the journal's data policy. If no structured public repository exists for a given data type, we encourage the provision of datasets in the manuscript as a Supplementary Document (see author guidelines under 'Expanded View' or in unstructured repositories such as Dryad (see link list at top right) or Figshare (see link list at top right).	Gb3 and Lyso-Gb3 levels in the mice reported in this study have been included as a supplementary document.
20. Access to human clinical and genomic datasets should be provided with as few restrictions as possible while respecting ethical obligations to the patients and relevant medical and legal issues. If practically possible and compatible with the individual consent agreement used in the study, such data should be deposited in one of the major public access-controlled repositories such as dbGAP (see link list at top right) or EGA (see link list at top right).	NA
21. Computational models that are central and integral to a study should be shared without restrictions and provided in a machine-readable form. The relevant accession numbers or links should be provided. When possible, standardized format (SBML, CellML) should be used instead of scripts (e.g. MATLAB). Authors are strongly encouraged to follow the MIRIAM guidelines (see link list at top right) and deposit their model in a public database such as Biomodels (see link list at top right) or JWS Online (see link list at top right). If computer source code is provided with the paper, it should be deposited in a public repository or included in supplementary information.	NA

G- Dual use research of concern

22. Could your study fall under dual use research restrictions? Please check biosecurity documents (see link list at top right) and list of select agents and toxins (APHIS/CDC) (see link list at top right). According to our biosecurity guidelines, provide a statement only if it could.	NA
